# Therapeutic potential of a newly isolated bacteriophage against multi-drug resistant *Enterococcus faecalis* infections: in vitro and in vivo characterization

**DOI:** 10.1186/s12866-025-03785-z

**Published:** 2025-02-20

**Authors:** Zienab Ali, Karim Abdelkader, Maha M. Abdel-Fattah, Ahmed Farag Azmy, Ahmed O. El-Gendy, Tarek Dishisha

**Affiliations:** 1 Department of Pharmaceutical Microbiology and Immunology, Faculty of Pharmacy, Beni-Suef University, Beni-Suef, 62511 Egypt; 2 Department of Pharmacology and Toxicology, Faculty of Pharmacy, Beni-Suef University, Beni-Suef, 62511 Egypt

**Keywords:** VRE, Vancomycin-resistant enterococcus, *Enterococcus faecalis*, Bacteriophage, In vivo animal model, Anti-biofilm

## Abstract

**Background:**

In nosocomial settings, vancomycin-resistant *Enterococcus faecalis* is a major health threat leading to increased morbidities, mortalities, and treatment costs. Nowadays, several approaches are under investigation to enhance the activity of or replace the traditional antibiotics. Bacteriophage therapy was sought as a potential approach for combating *E. faecalis* infections. The present study focuses on isolating and characterizing bacteriophage against clinical multi-drug resistant (MDR) *E. faecalis* strain Lb-1492. The phage stability, lytic activity, host-range, latent period, burst size, the ability to detach the pre-formed biofilm and destroy entrapped cells were investigated. The phage genome was purified, sequenced, and subjected to bioinformatics analysis for identifying and characterizing its features, as well as, the suitability for clinical application. Finally, the ability of the phage to rescue mice from deadly, experimentally induced *E. faecalis* bacteremia was evaluated.

**Results:**

A virulent phage was isolated from sewage water against a clinical MDR *E. faecalis* isolate. Morphological and genomic studies indicated that the phage belongs to the *Efquatrovirus* genus, with a long tail, icosahedral head and a linear double-stranded DNA genome of approximately 42.9 kbp. The phage was named *vB*_Efa_ZAT1 (shortly ZAT1). It demonstrated a shorter latent period and larger burst size than regular-tailed phages, and a characteristic stability over a wide range of pH and temperatures, with the optimum activity at pH 7.4 and 37 °C, respectively. Phage ZAT1 showed a narrow spectrum of activity and a characteristic biofilm disruption ability. The phage managed successfully to control *E. faecalis*-induced bacteremia in mice models, which was lethal within 48 h in the control group. An intraperitoneal injection of 3 × 10^8^ PFU of the phage solution given 1 h after the bacterial challenge was sufficient to save all the animals, completely reversing the trend of 100% mortality caused by this bacterium.

**Conclusions:**

Phage therapy can be a promising alternative to traditional antibiotics in the post-antibiotic era with a significant antimicrobial and antibiofilm activities against MDR *E. faecalis*.

**Supplementary Information:**

The online version contains supplementary material available at 10.1186/s12866-025-03785-z.

## Background

The misuse, overuse, and inappropriate disposal of antibiotics have led to the emergence of antibiotic-resistant pathogens [1], which have developed the ability to overcome a broad range of antibiotics. In recent decades, resistance mechanisms against most traditional antibiotics have been identified in some bacteria [2, 3]. In 2019, deaths attributable to antimicrobial resistance were estimated at 1.27 million worldwide [4]. In the United States, bacterial isolates resistant to at least one antibiotic account for over 70% of hospital-acquired infections, and in Japan, more than 50% of clinical *Staphylococcus aureus* isolates are multi-drug resistant (MDR) [5]. As a result, the rate of bacterial resistance is surpassing the rate of development of novel effective antibiotics [6, 7]. Hence, intervention is necessary to break this cycle [8, 9].

*Enterococcus faecalis* is a Gram-positive bacterium that can thrive in hot, salty, acidic, and alkaline conditions [10]. It is a natural resident of the intestinal tract of animals and humans and is recognized as an opportunistic pathogen [11]. In healthcare facilities in the U.S. between 2018 and 2021, *E. faecalis* ranked third (9%) among all pathogens reported for hospital-acquired infections in adults, and second (12.5%) to *Staphylococcus aureus* in nosocomial bloodstream infections reported in adult intensive care units [12]. It can cause various infections including infective endocarditis, urinary tract infections (UTIs), bacteremia, meningitis [13], periodontitis, neonatal sepsis, and wound infections, particularly in immunocompromised patients [14]. The microorganism can be transmitted from person to person through poor hygiene, contaminated food, and inadequately cleaned medical devices during surgery [11]. It is one of the most common bacterial species isolated from different types of wounds, such as diabetic foot ulcers, burns, and surgical sites [15–18]. It is also the third most frequently identified microorganism in surgical site infections [15–18]. Furthermore, it is associated with several hospital-acquired infections and exhibits resistance to many antibiotics, including vancomycin [19]. Due to its intrinsic and acquired resistance to various antibiotics, treating *E. faecalis* infections has become increasingly challenging [20, 21].

The resistance of *E. faecalis* to antibiotics can be attributed to several factors: (a) intrinsic resistance (efflux pump, cell wall modification, and presence of intrinsic β-lactamases), (b) acquired resistance (Horizontal gene transfer and mutation), (c) natural resistance to penicillin mediated by its penicillin-binding, (d) the ability to form a biofilm, (e) lower nutritional requirements and the ability to survive in the human body for extended periods without a source of nutrients, utilizing serum as a nutrient source, and (f) the ability to absorb folic acid from the surrounding environment [22–25].

With increasing bacterial resistance to traditional antibiotics, phage therapy has gained renewed attention for controlling human infections [26]. Bacteriophage therapy employs viruses as bioagents to target and destroy disease-causing bacteria. Due to its successful history of use in Eastern Europe, it is expected to become a practical alternative to antibiotics in the post-antibiotic era [27–29]. Several studies have demonstrated the superiority of phage therapy over conventional chemotherapy for certain applications [30–32]. Phage therapy offers several advantages [33], including (a) simple and cost-effective isolation of phages, (b) slower bacterial resistance compared to antibiotics [34], and (c) lower side effects on humans, among others. Moreover, bacteriophages has been successfully used synergistically with antibiotics for enhancing the latter’s activity [35–37]. Phage therapy however may trigger immune responses or bacterial overstress leading to acquired resistance [38]. Alternatively, phage-derived enzymes can be utilized instead of using the whole phage for treatment [33, 39].

In the present study, a lytic phage was isolated against MDR *E. faecalis* clinical isolate and subjected to morphological examination, phenotypic- and genotypic characterization. Later, the in vivo potential of this phage in controlling induced bacteremia in mice models was investigated.

## Methods

### Bacterial strains and culture media

A clinical MDR *E. faecalis* strain Lb-1492, previously identified and characterized [40] was used as a host microorganism for phage isolation. In addition, *Pseudomonas aeruginosa*, *Escherichia coli* ATCC 25,922, and clinical isolates of three previously characterized *Enterococcus faecium* isolates [41] were included for host range analysis. Regular bacterial cultivation was performed using LB broth which contained: 1% tryptone, 0.5% yeast extract, and 1% NaCl, at 37 °C. LB agar plates (LB supplemented with 2% w/v agar) were also used for bacterial cultivation. Bacterial strains were stored as 50% glycerol stock at -20 °C.

### Phage isolation, purification, and high titer preparation

Phage isolation was conducted using an enrichment technique [42] with the *E. faecalis* strain Lb-1492 clinical isolate as the host strain. Sewage samples (200 ml) were collected from the central sewage treatment station in Beni-Suef, Egypt, in sterile 50 ml Falcon tubes. The collected samples were centrifuged at 8000 ×g for 20 min at 4 °C and then filtered through a 0.45 μm syringe filter to remove particulate debris and bacterial and fungal contaminants. Next, 50 ml of the filtered supernatant was mixed with an equal volume of double-strength LB broth inoculated with 200 µl of exponentially grown host strain (*E. faecalis*) with an optical density of 0.6 at 620 nm. The mixture was incubated at 37 °C with shaking at 120 rpm for 18 h. Subsequently, the enriched filtrates were centrifuged at 8000 ×g for 20 min at 4 °C and sterilized through a 0.22 μm filter.

Screening for lytic phages was performed by spotting 10 µl of the filtrate on the surface of overlay soft LB agar (0.5% agar, Himedia, India) seeded with 100 µl of exponentially grown host strain (OD_620nm_ ~ 0.6). The plates were left to dry and then incubated at 37 °C for 16 h. To prepare a stock phage solution, lysis zones were cut using sterile razors, collected in sterile 900 µl sodium-magnesium (SM) buffer (10 mM Tris-HCl, 10 mM MgSO_4_, and 100 mM NaCl, pH 7.5), centrifuged at 8000 ×g for 20 min at 4 °C, and sterilized through a 0.22 μm filter.

Phage purification was carried out by seeding overlay soft agar with equal volumes (100 µl) of exponentially grown host strain mixed with serial dilutions of the previously prepared stock phage solution using a double-layer assay [43]. Individual plaques were then picked and suspended in 200 µl SM buffer. This step was repeated at least five times until obtaining morphologically similar plaques.

For high titer preparation, at least 2 lysis plaques obtained from spot assay were collected in 2 ml of SM buffer, left for 2 h at room temperature then centrifuged at 8000 ×g for 20 min at 4ºC and sterilized through 0.22 μm filter. Phage titer was counted using a standard double-layer assay [43].

### Transmission electron microscope (TEM)

Concentrated phage filtrate (10^10^ PFU/ml) was centrifuged at 15,000 *×*g for 3 h at 4ºC, washed twice with 0.1 M ammonium acetate (pH 7), and then suspended in SM buffer. Afterward, 5 µl of phage suspension was deposited on a formvar-coated grid and allowed to dry for 20 s at room temperature. Finally, the sample was stained with 2% uranyl acetate, then dried using filter paper and examined by transmission electron microscopy (TEM) at an accelerating voltage of 80 KV using a JEM-2100 (Joel LTD, Tokyo, Japan) microscope.

### Host range determination and stability evaluation of the phage

Host range determination of the isolated phage was conducted by spotting 10 µl of phage suspension (10^10^ PFU/ml) on *E. faecalis* strain Lb-1492 lawn using double–layer agar method. Then, the plates were left for drying and incubated at 37ºC for 18 h. A panel of 7 bacterial strains was included for host range determination.

Phage stability was evaluated against a wide range of temperature (4ºC – 75ºC) and pH (4–9). For thermal stability, phage stock (10^10^ PFU/ml, in SM buffer, pH 7.5) was incubated in a specified temperature for 1 h with sampling intervals of 10 min. After each interval, the remaining infective phages were counted using a double-layer technique against *E. faecalis* strain Lb-1492 as a bacterial lawn. Similarly, pH stability was screened by diluting phage stock in SM buffer with respective pH for 1 h at 25ºC. Then, the phage titer was counted at the end of the incubation period (1 h) using a double-layer assay.

### Adsorption and one-step growth curve assays

Adsorptions and one-step growth curve assays were conducted as described previously [44] with minor modifications. For adsorption assay, exponentially grown *E. faecalis* in LB broth (OD_620nm_ = 0.6) was mixed with phage stock prepared in SM buffer (pH 7.4, with and without 10 mmol/l CaCl_2_) to obtain a final MOI of 0.5. Then, the mixture was incubated at 37ºC for a period of 20 min. Hundred microliters were sampled at 2 min intervals over a period of 10 min followed by two sampling steps at 5 min intervals. Withdrawn samples were then 10-fold diluted in cold LB broth, centrifuged at 8000 ×g for 5 min, and free phages in the supernatant were counted using a double-layer assay. The experiment was conducted as three independent replicates.

For one-step growth curve, 10 ml of exponentially grown *E. faecalis* was infected with the isolated phage to have a final MOI of 0.01. Afterward, the mixture was incubated for 5 min at 37ºC to allow phage adsorption, centrifuged at 8000 ×g for 5 min then the pellets were re-suspended in 10 ml fresh LB broth. Samples were taken at 5 min intervals for the first 30 min followed by 10 min sampling till 1 h. The experiment was performed in triplicates and the curve was constructed by plotting PFU/ml against time.

### Antibacterial and anti-biofilm activity assays

The effect of the isolated phage on *E. faecalis* strain Lb-1492 growth was evaluated as reported earlier [45]. For this, an exponentially grown host strain (OD_620 nm_=0.6) was diluted in fresh LB medium to obtain a final bacterial cell count of 10^7^ CFU/ml. Subsequently, 100 µl of the culture was dispensed in wells of 96-well microtiter plates and mixed with 100 µl of ten-fold serial dilutions of the phage (in LB broth) to cover MOI range of (0.1–100). Finally, the plates were incubated at 37ºC and optical density (OD) was recorded at 1 h intervals over a total period of 12 h.

The anti-biofilm potential of the isolated phage was assessed using two techniques: the crystal violet (CV) assay to evaluate the phage’s ability to disrupt a pre-formed biofilm matrix, and the viable count assay to determine its effect on bacteria embedded within the established biofilm [46].

In crystal violet assay, *E. faecalis* strain Lb-1492 was grown at 37ºC for 24 h in tryptone soya broth (TSB) supplemented with 1% glucose. Afterward, the culture was 100-fold diluted in a fresh TSB-glucose, dispensed with volumes of 200 µl in 96-microtiter plate wells, and incubated at 37ºC for 48 h in a static condition. After incubation, the free bacteria were decanted whereas the formed biofilm was gently washed twice with PBS (8 g/l NaCl, 0.2 g/l KCl, 1.42 g/l Na_2_HPO_4,_ and 0.24 g/l KH_2_PO_4_) and then incubated with phage at MOIs of 0 (control), 1, 10, and 100 for 2 h at 37ºC. Next, the added phage suspensions were discarded, and the residual biofilm was stained with 1% crystal violet for 15 min at room temperature. The stained biofilm was then washed gently with PBS and the stain was dissolved with 150 µl of 95% ethanol and the optical density was measured at 570 nm. Controls were conducted by replacing phage suspension with PBS buffer. The experiment was conducted as five independent triplicates.

For counting biofilm-embedded bacteria, 48 h age biofilms were formed and treated with different phage concentrations suspended in PBS buffer as earlier. The phage solution was discarded and 100 µl of PBS was added to each well. The wells were subjected to sonication for 1 min using a water bath sonicator (Sonix TV ss-series ultrasonicator, Sonix IV Ultrasonic Cleaning Systems, North Charleston, SC, USA). The resulting suspension was serially diluted in PBS and then the viable cells were counted in 10 µl of properly diluted suspension after cultivation on LB agar at 37 °C for 24 h. The colony forming units were determined using the following equation and compared to the control (PBS without phage):

$${\rm{CFU/ml }} = N \times 1000{\rm{ }}/V \times D$$

Where N is the number of colonies, V is the volume of the sample (10 µl) and D is the dilution factor.

### Phage genomic extraction

Phage genomic DNA was extracted from filter sterilized high titer phage stock (10^10^ PFU/ml in SM buffer with pH 7.4) using phenol/chloroform/isoamyl alcohol (25:24:1) protocol [47]. Firstly, phage stock was incubated with DNaseI (final concentration of 1 µg/ml; Sigma-Aldrich, UK) and 1 µg/ml of RNaseA (Sigma-Aldrich, UK) for 30 min at 37ºC to break down bacterial genomic material. Afterward, the viral capsid was breached by incubation with 20 mM ethylene diamine tetra-acetic acid (EDTA), 50 µg/ml proteinase K and 0.5% sodium dodecyl sulfate (SDS) at 56ºC for 1 h. After two rounds of phenol/chloroform/isoamyl alcohol extractions, DNA in the aqueous layer was precipitated by the addition of three volumes of 95% ethanol and one-tenth volume of 3 M sodium acetate pH 5 followed by 10 min incubation in ice. Then, DNA was pelted, washed twice using cold 70% ethanol, and re-suspended with 50 µl deionized water. Genomic DNA was quantified spectrophotometrically using NanoDrop™ 2000/2000c (Thermo Fisher Scientific, MA, USA).

### Genome sequencing, annotation, and bioinformatics analysis

Phage genomic sequencing was performed using the Illumina MiSeq NGS DNA sequencing platform (LGC, Berlin, Germany). Subsequently, the obtained raw data were paired and trimmed to remove adaptors using the BBDuk trimmer plugin of Geneious^®^ 7.1.3 software (https://www.geneious.com) with a Q value of 30. The trimmed sequence was then de novo assembled using Geneious de novo assembly with default settings. Rapid Annotation subsystem Technology (RAST, version 2.0) [48], Prokka version 1.12 [49], and Glimmer 3 [50] platforms were used for automatic annotations. The annotated coding genes were further confirmed using BLASTx analysis [51] against a non-redundant protein database, whereas conserved domains within encoded proteins were screened using NCBI Conserved Domain Database (CDD) [52]. Functional analysis of predicted proteins was investigated using InterPro [53], while structural similarity with other homologous proteins was analyzed using HH Pred [54]. The possible transmembrane domains within putative holins and lysins were detected using DeepTMHMM [55]. The presence of t-RNA was screened using tRNAscan-SEv.2.0 [56]. Genomic visualization was performed using CGview online server [57]. Phage lifestyle and existence of virulence genes or antimicrobial resistance markers were predicted using phage leads-based machine learning approach [58].

For genomic comparison, BLASTn analysis was performed to detect phages with the highest similarity at the nucleotide sequence level. Average nucleotide identity (ANI) based on BLAST+ (ANIb) was also calculated by JSpeciesWS [59] for phages with genomic similarity > 90%. Moreover, the pairwise intergenomic similarities of these phages to the isolated phage were computed by the Virus Intergenomic Distance Calculator (VIRIDIC) [60]. Viral Proteomic Tree (ViPTree) was used to generate a proteomic tree of the phage genome based on genome-wide sequence similarities computed by tBLASTx [61].

### In vivo study of phage therapy against bacterial infection in mice

#### Animals

Adult male Swiss mice, six weeks old and weighing 22–30 g, were procured from El-Nahda University’s Central Animal House in Beni-Suef. All animals were fed antibiotic-free food and had free access to water, housed at 25 ± 2 °C in a 12/12-hour light/dark cycle. During the study period, animals were properly cared for.

#### Bacterial preparation and determination of minimal lethal dose (LD100)

Bacterial inoculum was created by incubating *E. faecalis* in 300 ml TSB overnight at 37 °C without shaking, followed by centrifugation at 10,000 rpm and 4 °C for 10 min. The pellet cell was washed using 300 ml of sterile saline solution, centrifuged at 10,000 rpm and 4 °C for 10 min, and then re-suspended in 3 ml of saline. The bacterial concentration was adjusted through dilution with saline solution and then the viable count was determined. To determine the lethal dose causing 100% lethality (LD_100_) of *E. faecalis* culture, 100 µl doses ranging from 10^2^ to 10^10^ CFU/ml were injected intraperitoneally. Mice injected with buffered saline, PBS (pH 7.2) served as a control. Mice inoculated with bacteria were scored on a scale of 5 to 0, based on the progression of the disease as reflected by several clinical signs [62].

The scoring system was indicated as 5: Normal (healthy); 4: slight illness, defined as lethargy and ruffled fur; 3: moderate illness, defined as severe lethargy, ruffled fur, and hunched back; 2: severe illness, with the above signs plus exudative accumulation around partially closed eyes; 1: a moribund state; 0: death [63].

#### Preparation of phage for in vivo studies

For the mouse rescue experiment, the purified phage sample was dialyzed against PBS (three times each for 60 min with intermittent change of the PBS, and then left on a shaker overnight). The phage’s titers (PFU/ml) were then determined.

#### Treatment of E. faecalis-induced bacteremia using phage

Mice (6–7 weeks old, 22–30 g) were used in the present study. The phage preparation in PBS was diluted to the following multiplicities of infection (MOI): 10, 1 and 0.1. Mice were injected intraperitoneal (IP) with 200 µl of the minimum lethal bacterial dose (in total 36 mice, 6–7 weeks old; four groups, each group 6 mice, Table S1). About 60 min after application of the lethal bacterial dose, 200 µl from each MOI of phage solution were injected to each group of mice, respectively. Subsequently, 100 µl of blood samples were collected from the retro-orbital plexus at 1, 3, 5, 24, 48, and 72 h intervals and were directly added to 0.05 M EDTA [64]. Bacterial count in the collected samples was done by two methods (a) direct dropping the blood on nutrient agar, and (b) making serial dilution in saline then making viable count and incubating at 37 °C. Three mice were sacrificed by cervical dislocation at day 7 and day 11, respectively. The heart was collected aseptically and preserved in 10% formalin for histopathological study.

#### Histopathological examination

The heart tissues were compared with normal, in the case of phage therapy and bacterial challenge. The tissues were initially dehydrated using rising concentrations of alcohol for dehydration (70–100%) and then conserved in 10% formalin. The tissues are then immersed in paraffin wax, cut, and stained with hematoxylin and eosin [64].

### Statistical analysis

The data from the in vitro experiments were analyzed using Microsoft^®^ Excel Analysis ToolPak. A one-way analysis of variance (ANOVA) was first conducted to assess the significance of the mean values of the dataset, followed by a Student’s t-test to determine significant differences between individual treatment means, with a p-value threshold of < 0.05, unless otherwise specified.

### Accession number

The phage vB_Efa_ZAT1 full genome sequence was deposited in the NCBI GenBank database under accession number PP438801.

## Results

### Isolation and propagation of phage against MDR *E. faecalis*

**Fig. 1:**
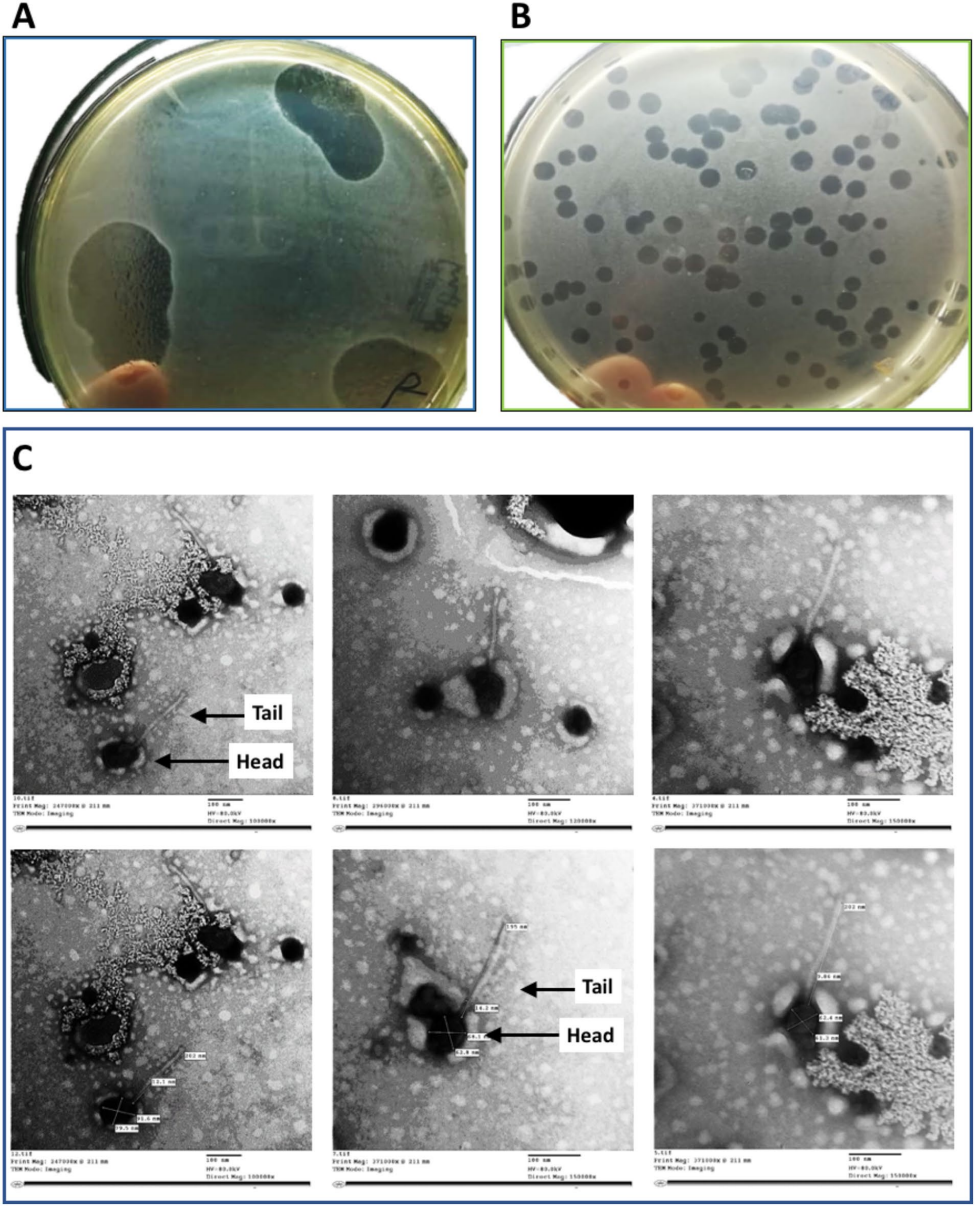
Spot test, plaque assay and TEM of *Enterococcus* phage vB_Efa_ZAT1. (**A**) Spotting phage on soft agar overlay containing host bacterial suspension. (**B**) The plaque assay test after several rounds of purification and selection. The plaque was purified on double-layer agar plates. (**C**) Transmission Electron micrographs of phage ZAT1 showing phage particles as an icosahedral head (79.5 nm in width and 91.6 nm length) attached to a long tail (202 nm in length and 12.1 nm in width)

A phage was isolated from a wastewater sample, using *E. faecalis* strain Lb-1492 clinical isolate as a host. The phage exhibited lytic activity against the host strain, forming clear zones when spotted on the host bacteria and producing clear plaques when added to an overlay soft agar layer (Fig. 1A). The purified phage produced a circular clear zone of approximately 2 mm diameter on the double-layer agar plate (Fig. 1B). The phage was given the name vB_Efa_ZAT1 (Shortly ZAT1).

### Morphology features

Morphological examination of phage ZAT1 using a transmission electron microscope revealed that it had a clear icosahedral head measuring 79.5 nm in width and 91.6 nm in length (Fig. 1C). The head was attached to a long tail measuring 202 nm in length and 12.1 nm in width. These features suggest a classification of the phage within the *Efquatrovirus* genus.

### Thermostability, pH stability and effect of Ca^+ 2^ ions on phage adsorption

**Fig. 2:**
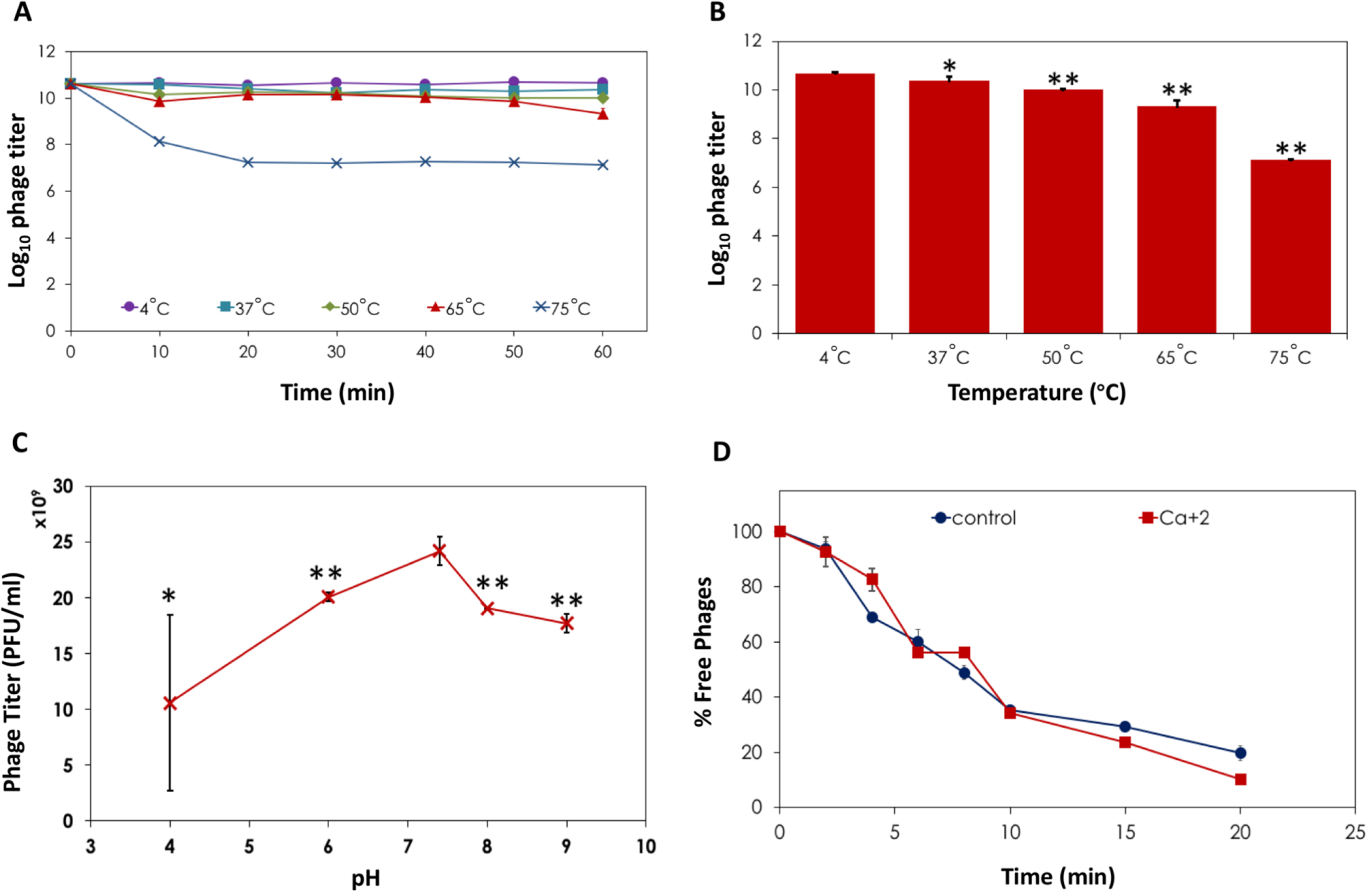
Thermal and pH stability and effect of Ca^+ 2^ on *Enterococcus* phage ZAT1 adsorption. (**A**) Time killing curve showing change in log_10_ PFU/ml over 1 h incubation at different temperatures. (**B**) Log_10_ the residual PFU/mL after 1 h incubation at different temperatures. Asterisks indicate a significant difference from that at 4 °C (**: *P* < 0.05) and (*:*P* < 0.1). (**C**) Changes in the phage titer as determined by plaque assay after the incubation of phage solution in SM buffer at different pH values for 1 h. Asterisks indicate a significant difference from that at pH 7.4 (**: *P* < 0.05). (**D**) Effect of calcium ions on adsorption of phage ZAT1 to host cells. Percentage of free phage = (N_T_/ N _o_) *100, (1) where N_0_ is the PFU/ml of phages at T = 0 min and N_T_ is the PFU/ml at time T

The thermal stability test showed that phage ZAT1 was highly stable at 4 °C, 37 °C, 50 °C and 65 °C. However, its stability decreased significantly when exposed to 75 °C for 60 min (Fig. 2A, B). The average initial phage titer was 40 × 10^9^ PFU/ml which was stable around the same titer upon exposure to 4 °C for 60 min. The titer was slightly reduced to 24.33 × 10^9^, 10.03 × 10^9^ and 1.33 × 10^9^ PFU/ml after incubation for 60 min at 37 °C, 50 °C and 65 °C, respectively. However, the titer dropped sharply to below 1 × 10^7^ PFU/ml after incubation for 20 min at 75 °C (equivalent to 3.5 log reduction) and remained stable until 60 min (Fig. 2A, B).

Regarding pH stability, phage ZAT1 exhibited good stability over a wide range of pH values for 60 min (Fig. 2C). It maintained a high titer (> 1 × 10^10^ PFU/ml) after incubation at pH values ranging from 4 to 9 for 60 min, with the optimum stability observed at pH 7.4.

The effect of calcium ions on the adsorption of phage ZAT1 was evaluated by supplementing 10 mM of CaCl_2_ to the phage and host bacteria. The results showed that calcium ions had a negligible effect on phage adsorption, as there was no significant difference compared to the control without calcium ions supplementation (Fig. 2D).

### Phage ZAT1 host range

To determine the host range of phage ZAT1, six strains of different bacteria were tested by spotting the phage on an agar plate. The phage showed specificity to *E. faecalis*, the host bacterium used for isolation, as it did not exhibit any activity against other tested strains, including *E. faecium*, *E. coli* and *P. aeruginosa*. Only slight activity was observed against *E. faecium* 65 strain (Table 1).

**Table 1:** Determination of host range of ZAT1 phage

Clinical isolate	Source of strain	The growth after application of phage
*Enterococcus faecalis* strain Lb-1492	Urine	+++
*Enterococcus faecalis*	Stool	
*Enterococcus faecium 43*	Urine	
*Enterococcus faecium 65*	Urine	+
*Enterococcus faecium 43’*	Urine	
*Pseudomonas aeruginosa*	Ear pus	
*Escherichia coli*	Urinary tract infection	

+++: Significant activity (clear plaques, high titers), ++: Moderate activity (smaller plaques, moderate titers), +: Slight activity (tiny/turbid plaques, low titers), -: No activity (no plaques, no lysis)

### Lytic activity and one step growth curve of phage ZAT1

**Fig. 3:**
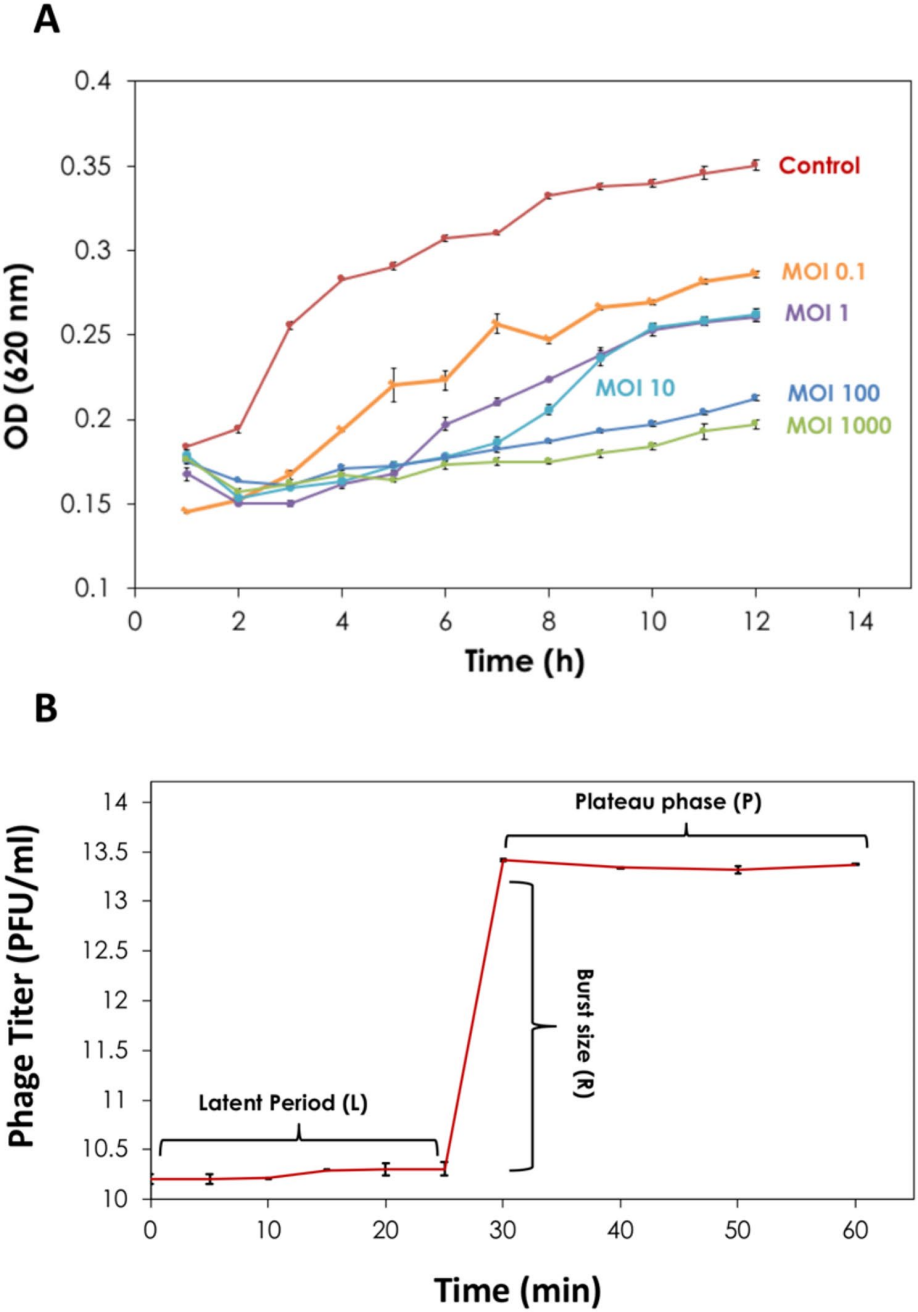
Time-killing curve and the one-step growth curve of *Enterococcus* phage ZAT1. (**A**) Time killing curve analysis done in presence of phage ZAT1 against *Enterococcus faecalis* strain Lb-1492 using different MOIs (0 control, 0.1, 1, 10, 100 and 1000) over 12 h. (**B**) One-step growth curve of phage ZAT1 at MOI of 0.1 showing a typical tri-phasic pattern. The latent period was estimated as 25 min, and the burst size was calculated to be ∼1263 PFU/cell

The kinetics of growth inhibition of the clinical *E. faecalis* host strain by phage ZAT1 at different multiplicities of infection (MOIs) were investigated. Without phage treatment, the *E. faecalis* isolate exhibited an initial lag phase of 1 h, followed by a logarithmic phase where the density (OD_620_ nm) increased sharply from 0.19 to 0.26 between the 2nd and 3rd hours (*µ*_*max*_ = 0.27 1/h). The density then increased slowly from 0.26 to 0.35 over 8 h (*µ* = 0.04 1/h). However, when treated with phage ZAT1 at various MOIs (0.1, 1, 10, 100, and 1000), the OD_620_ nm values were consistently lower than that of the positive control (*p* < 0.05), indicating efficient inhibition of bacterial growth by the phage. Figure 3A presents the increase in the optical density in case of the control (without phage) and in case of phage treatment with different MOIs.

The one-step growth curve of phage ZAT1, propagated on the host strain in LB medium, revealed that the latent period was approximately 25 min, and the rise period was approximately 30 min (Fig. 3B). The average burst size was estimated to be 1263 PFU/cell.

### Anti-biofilm activity

**Fig. 4:**
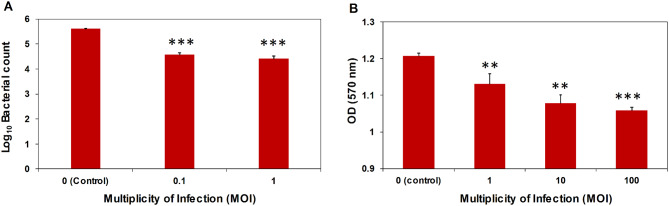
Antibiofilm activity of phage ZAT1 against *E. faecalis* strain Lb-1492. The activity was estimated as (**A**) Log reduction in bacterial viable count after releasing the bound cells using sonication. (**B**) Reduction in biomass in the biofilm as determined using the crystal violet staining method. Asterisks indicate a significant difference from the control (***: *P* < 0.01, **: *P* < 0.05). Experiments were run in three replicates and the presented data are the average of these replicates + standard deviation

The anti-biofilm activity of phage ZAT1 was investigated. Application of the phage to established biofilms significantly reduced the bacterial viable count compared to the untreated control (Fig. 4A). The crystal violet staining method was employed to evaluate the biofilm disruption ability of the phage solution, which showed a significant (*P* < 0.05) decrease in biofilm staining for the phage-treated wells compared to the control (Fig. 4B).

### General features of phage genome

**Fig. 5:**
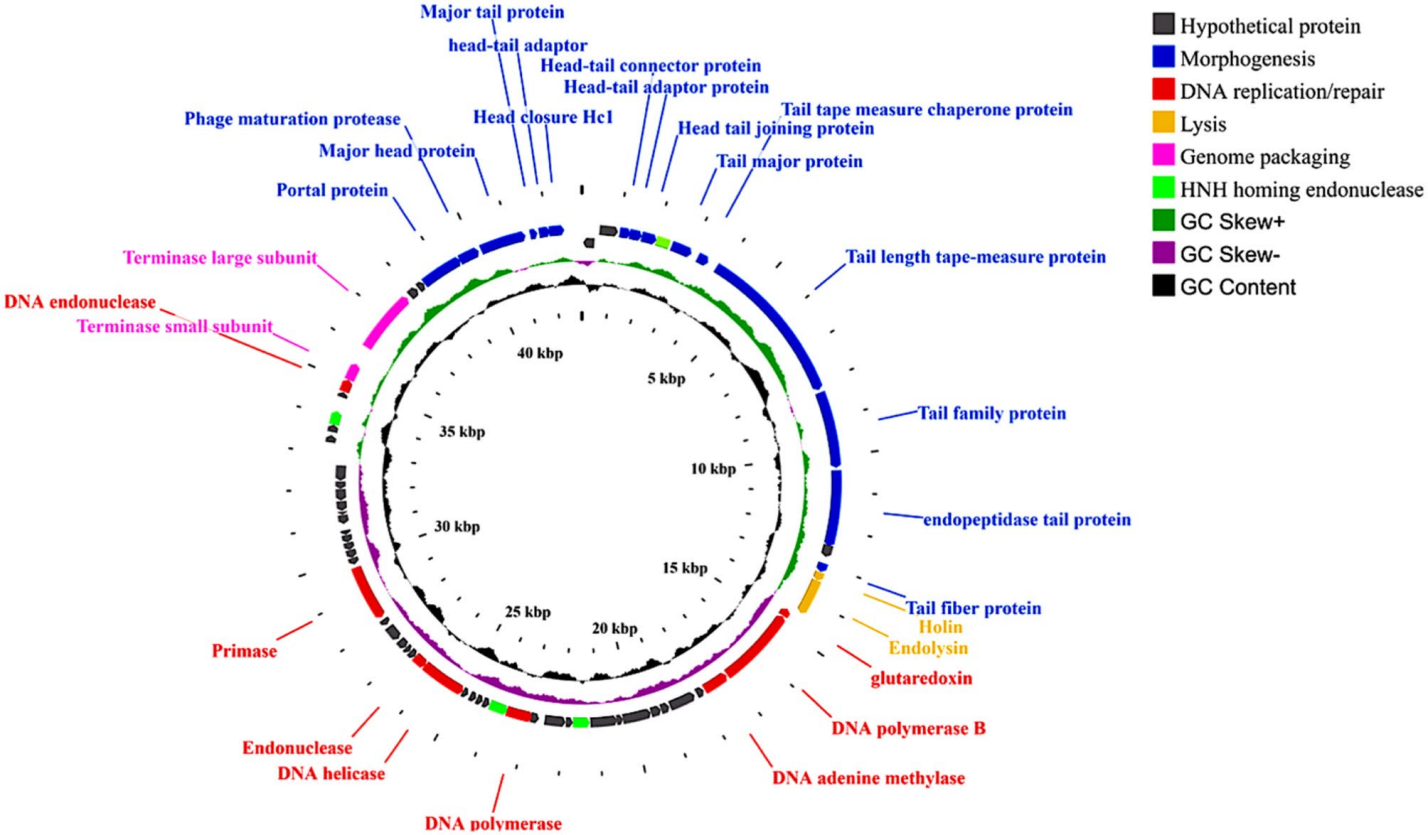
Genotypic characterization of phage ZAT1

*Enterococcus* phage ZAT1 has a linear double-stranded DNA genome with a total length of 42,928 bp and GC content of 34.8% (Accession number: PP438801). The assembled genome showed no t-RNA, resistance genes or temperate phage markers (integrases, excisionases, recombinases, transposases) suggesting its suitability for phage therapy. Phage genomic annotation predicted 67 open reading frames (ORFs; Table S2: see Additional file 1). The reading frames are disturbed on both strands of the phage genome and display ATG start codon except for *orf29* (TTG) and *orf45* and *orf63* (GTG; Table S2: see Additional file 1). Functional analysis of the phage genome predicted 39 (58%) genes with unknown assigned functions (hypothetical proteins; Fig. 5 and Table S2: see Additional file 1). The remaining reading frames are assigned with functions related to morphogenesis (15, 22.3%), DNA replication\repairing (8, 11.9%), host lysis (2, 2.9%), and genomic packaging lysis (2, 2.9%) (Fig. 5 and Table S2: see Additional file 1). Three HNH homing endonucleases (*orf25*, *orf30*, *orf55*) were detected within the phage genome as separate genes without interrupting adjacent genes (Fig. 5 and Table S2: see Additional file 1).

The morphogenesis module comprises the largest part of the phage genome spanning ~ 20 kbp and is either related to head, tail, or connector proteins (Fig. 5 and Table S2: see Additional file 1). A tail fiber associated with C-terminal endopeptidase (*orf11*) and tail length tape-measure protein with lyz_like_superfamily (*orf9*) conserved domain were observed among morphogenesis modules. Interestingly, no depolymerases were found within the phage genome.

The observed DNA replication coding genes are DNA polymerase (*orf29*), helicase (*orf35*), and primase (*orf42*) whereas DNA repairing genes are DNA adenine methylase (*orf18*), endonuclease (*orf36*) and endo-deoxyribonuclease (*orf57*). Phage ZAT1 adopts a DNA packaging system with two subunits, large (*orf59*) and small (*orf58*) terminase subunits. The phage utilizes two-component lysis system including non-overlapping holin-endolysin system without using spanin. Functional analysis of the predicted lysin showed a putative amidase activity, cleaving the bond between N-acetyl muramic acid and L-alanine with the peptidoglycan meshwork. The predicted holin (*orf14*) shows two transmembrane domains with C-in N- in topology indicating class II holin.

BLASTn analysis for the phage genome displayed more than 50 phages with nucleotide similarity up to 92.14% and query coverage up to 91% (Table S3: see Additional file 1). All similar phages have genome sizes ranging from 21,115 to 41,712 and belong to *Efquatrovirus* genus under class *Caudoviricetes*, family *Siphoviridae* (tailed phages). ANI analysis with the top similar phages (Table S3: see Additional file 1) showed values less than 95%, a cut-off value for species delineation, suggesting ZAT1 as a new species of *Efquatrovirus* genus.

CG viewer representation of phage ZAT1 genome. The outer ring represents the predicted ORFs (arrows) of the phage genome with different colors representing the different modular functional groups. The middle and inner rings illustrate the GC skew and GC content, respectively. The ZAT1 genome reveals a typical modular organization with DNA replication/transcription, morphogenesis, host lysis and DNA packaging genes, apart from a region dominated by hypothetical genes.

### Phylogenetic analysis

**Fig. 6:**
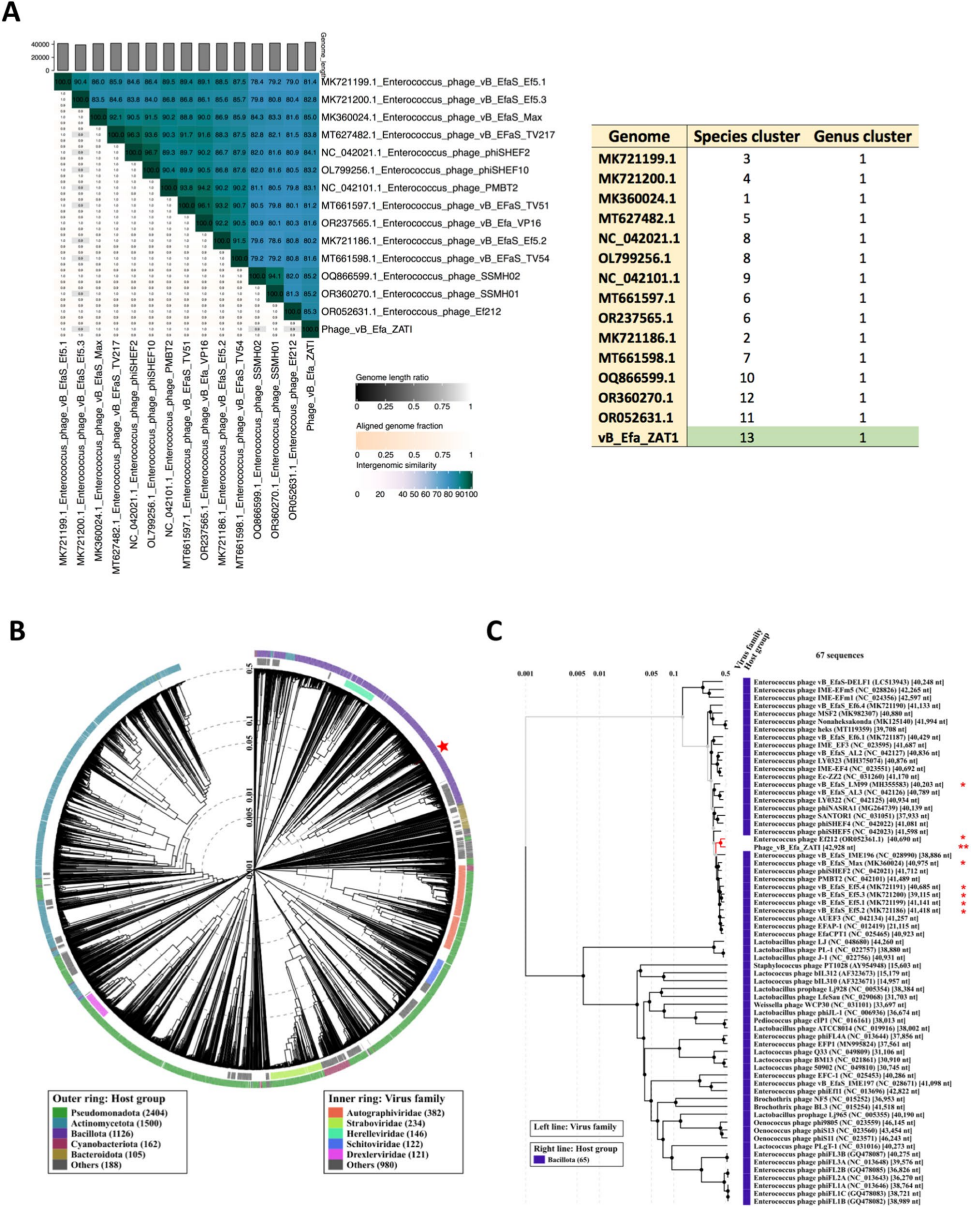
Intergenomic Similarity and proteomic tree generated of phage ZAT1. (**A**) Heatmap showing ZAT1 with genomic-close *Enterococcus* phages obtained using VIRIDIC [60]. (**B**) Circular proteomic tree of phage ZAT1, top BLASTn hits, and related phages of RefSeq genomes as generated by VIPTree [61]. (**C**) Rectangular tree represents a subset of the closely related phages from circular tree. (*) indicate unclassified *Efquatrovirus*, while (**) indicate phage ZAT1 (Query)

VIRDIC was employed to compute the intergenomic similarity of phage ZAT1 and the top-matched phages, and *Enteroccous* phages Ef212, SSMH01, SSMH02 and vB_EfaS_Max were within the genus threshold (85.3–85.0% intergenomic similarity). Therefore, phage ZAT1 and the other phages were clustered into the same genus but different species (Fig. 6A).

**Fig. 7:**
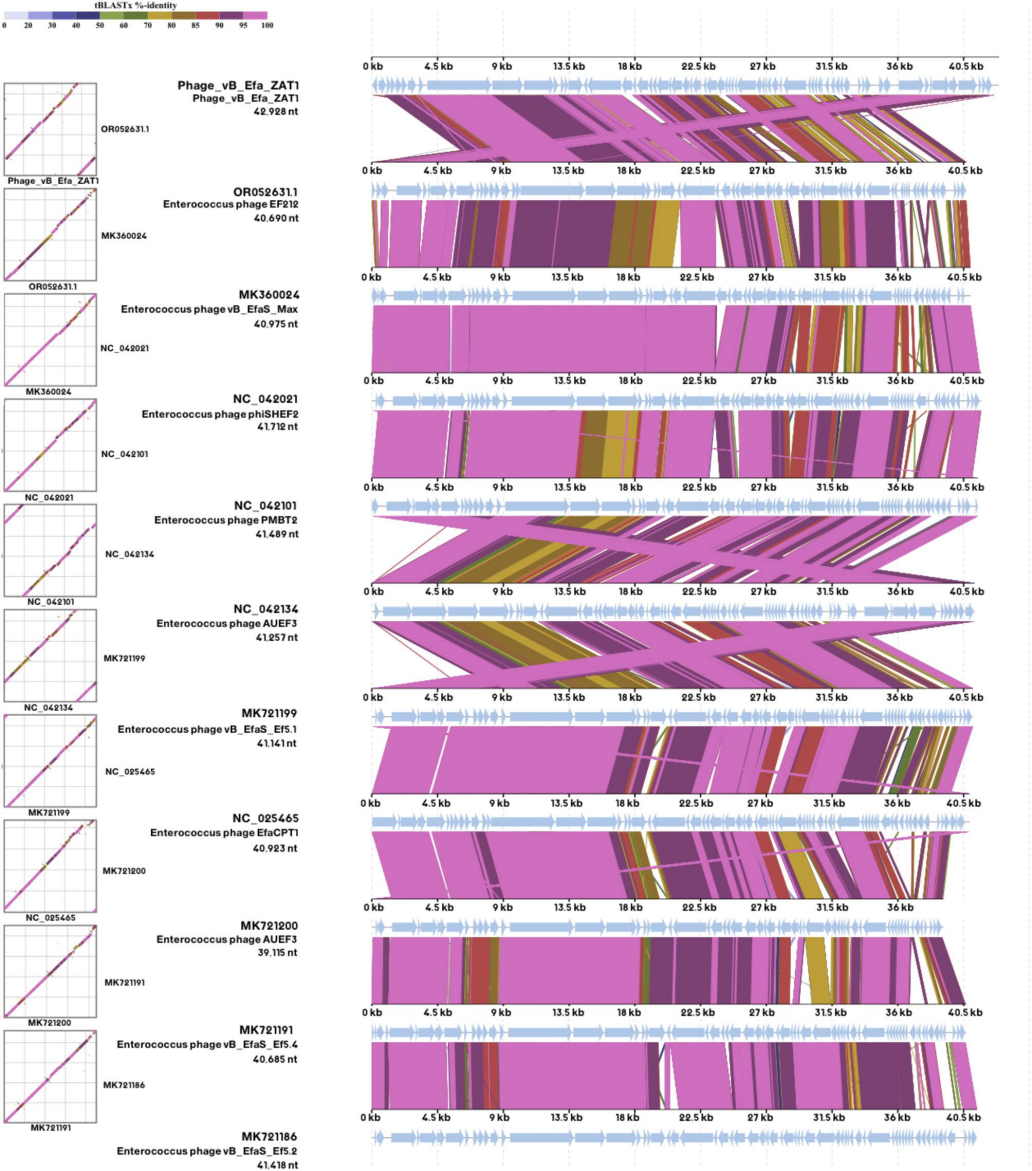
Whole-genome alignment and comparison of phage ZAT1 and closely related phages. Determined using the Dynamic Genomic Alignment server (DigAlign) [65]

Moreover, the proteomic tree inferred that phage ZAT1 is closely related to *Enterococcus* phage Ef212. The proteomic tree grouped the phage ZAT1 with unclassified *Efquatrovirus* in a separate clade from *Efquatrovirus* of the class *Caudoviricetes* (Fig. 6B, C). The genomes of the closely related phages (mainly ViPTree score *SG* > 0.78) were aligned and compared to ZAT1 (Fig. 7). The whole genome comparison highlighted the differences between the genomes, and particularly, between phage ZAT1 and the closest phage (*Enterococcus* phage Ef212, ViPTree score *SG* = 0.87).

### Toxicity testing of phages in mice

Absence of toxicity is an important criterion in phage therapy. The toxicity of phage preparation was investigated against mice. The experiment demonstrated the survival of the whole set of mice (without *E. faecalis* infection) giving an arbitrary scale with a score of 2 indicating the non-toxic nature of the applied phage [64]. The phage’s safety may be attributed to the absence of any microbial contamination in the phage suspension after dialysis so that it could be administered safe enough to compromise mice and thus was considered for further in vivo study.

### In vivo phage therapy against MDR *E. faecalis* isolate infection in mice

A mouse model was used to test the efficacy of phages in preventing/treating *E. faecalis*-induced bacteremia in mice [66]. The LD_100_ of MDR *E. faecalis* clinical isolate for IP dose was determined to be 10^9^ CFU/ml. This dose was found to be sufficient to cause bacteremia in mice, resulting in 90–100% mortality within 48–72 h of infection, correlating with McVay and coworkers’ findings [17]. One possible explanation for such high mortality rates is the rapid spread of disease.

The change in bacterial viable count in blood was evaluated in a mouse-type bacteremia after IP injection with *E. faecalis*. The evaluation was done at different time intervals over a period of 72 h (1, 3, 5, 24 and 72 h). A single IP injection of ZAT1 phage at different MOIs (10, 1, and 0.1) was found effective for controlling *E. faecalis*-induced bacteremia in the mice. The intraperitoneal route of administration of phage preparation was chosen because it provided the most significant protection (100%) when compared to other routes.

**Fig. 8:**
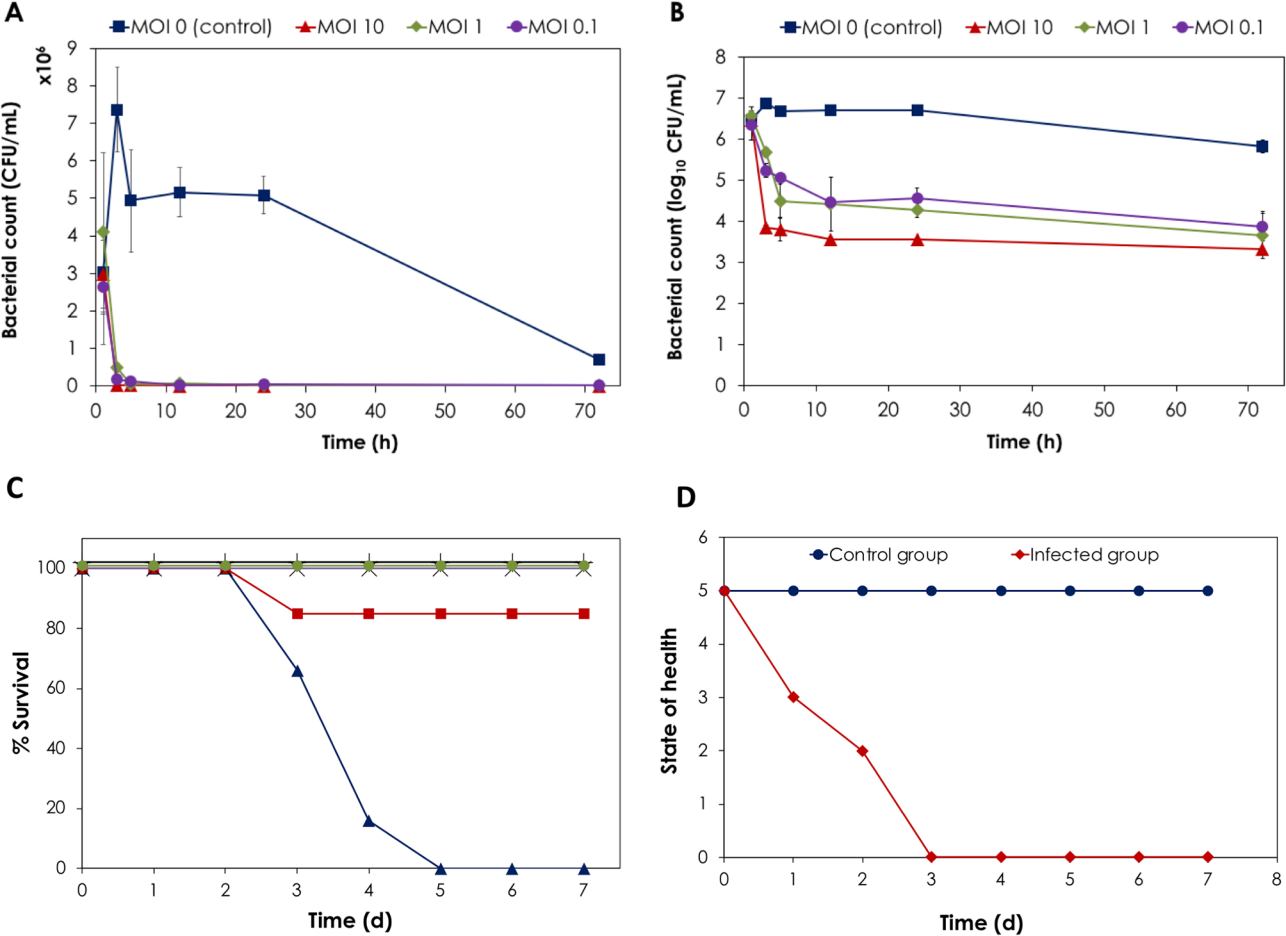
In vivo study of phage ZAT1 in mouse model. (**A**) Viable count of *E. faecalis* in mice blood samples at different time intervals after injection of the infective microorganism with and without injection of different MOIs of phage solution after 1 h. (**B**) The change in log_10_* E. faecalis* count in mice blood samples at different time intervals after injection of the infective microorganism with and without injection of different MOIs of phage solution after 1 h. (**C**) Survival rate of negative control (+), positive control (infection) (▲), treated group at MOI = 10 (◼), treated group at MOI = 1 (×), treated group at MOI = 0.1 (⬤). (**D**) The state of health score: 5: normal health; 4: slight illness, defined as lethargy and ruffled fur; 3: moderate illness, defined as severe lethargy, ruffled fur, and hunched back; 2: severe illness, with the above signs plus exudative accumulation around partially closed eyes; 1: a moribund state; 0: death

Figure 8A and B present the changes in the bacterial viable count and log bacterial count in blood samples collected from infected mice and phage-treated mice at different MOIs. It is observed that bacterial count increased in the control experiment from 3.04 × 10^6^ CFU/ml to 7.37 × 10^6^ CFU/ml within the initial 3 h and then stabilized around 5.06 × 10^6^ CFU/ml over the subsequent 21 h and then reduced to 0.69 × 10^6^ CFU/ml after 72 h. When the phage solution with MOI of 10 was applied 1 h after induction of bacteremia, the bacterial count was reduced significantly to below 6.9 × 10^3^ CFU/ml within the initial 3 h (equivalent to 2.6-log reduction) and was stable at 3.6 × 10^3^ CFU/ml between 12 and 24 h and reached a minimum of 3.2-log reduction (2.0 × 10^3^ CFU/ml) after 72 h. Both MOIs of 1 and 0.1 showed a comparable behavior, however, the reduction ranged between 2.8-log reductions in the former and 2.5-log reductions in the latter.

Moreover, the treatment with phage ZAT1 has successfully rescued the mice from death by *E. faecalis*-induced bacteremia compared to control group without phage treatment. This was observed as 100% survival rate for mice treated with phage solution at MOIs of 1 and 0.1, respectively, and 80% for phage solution with MOI of 10 (Fig. 12A). The non-treated control group showed a reduction in survival rate from 100% during the initial 2 days to 60% on the third day, 20% in the fourth day and 0% thereafter. The survival rate of control mice without any bacterial or phage treatment was 100% confirming that the death results mainly from *E. faecalis*-induced bacteremia. The state of health was also followed which showed a reduction in the scoring system from 5 to 0 for the infected group compared to the control group (Fig. 12B).

### Histopathology

**Fig. 9:**
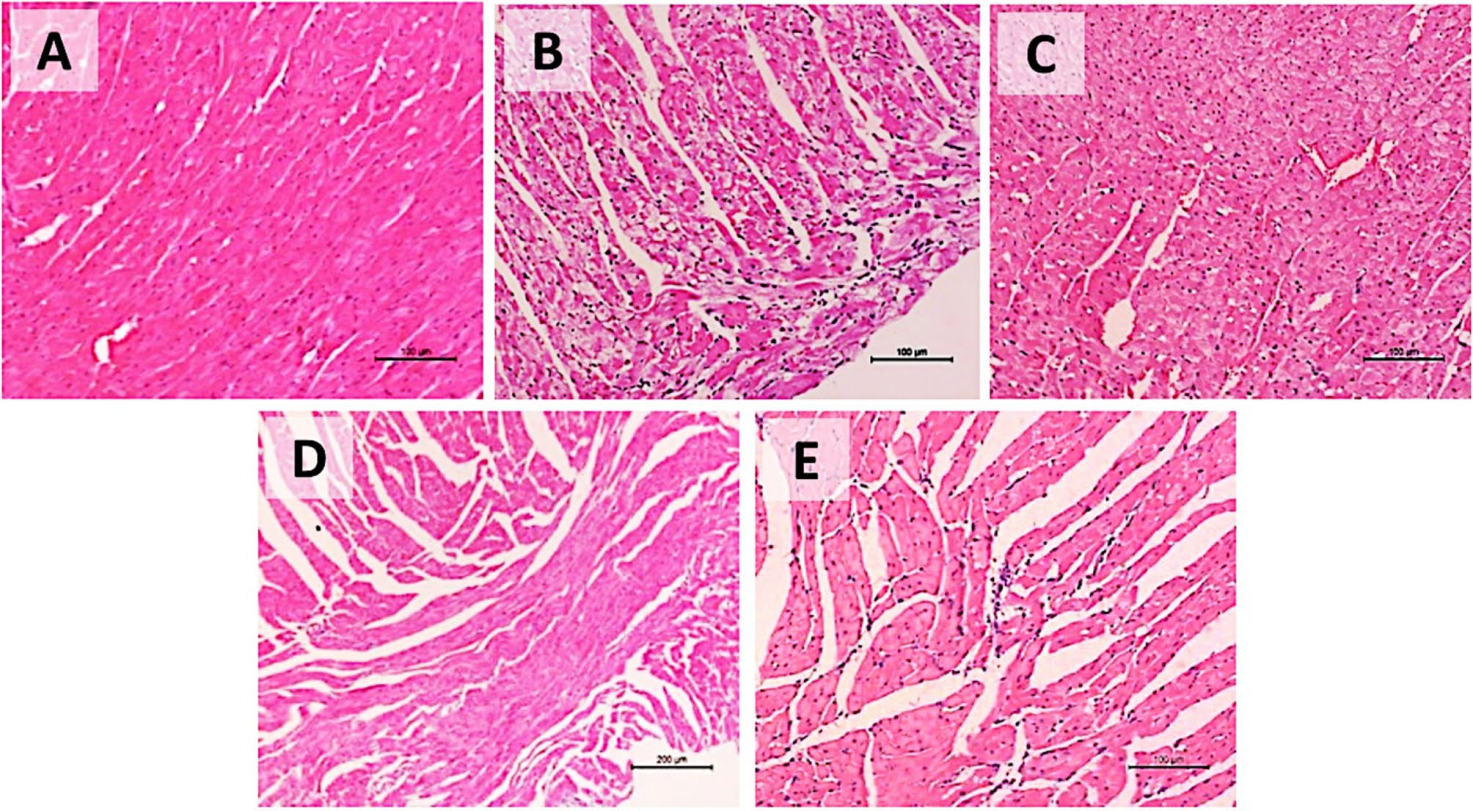
Photomicrographs of cardiac tissues showing histopathological features of normal, infected, and treated mice. (**A**) Normal (negative control), displayed a normal architecture of the myocardium, as myocardium forms a thick middle layer between the outer (pericardium) and inner (endocardium) layers of the heart wall, with blood supplied via coronary circulation. (**B**) Positive (infected mice): showed degenerative changes (necrosis) and leucocyte infiltration (in sever state). (**C**) Treated group at MOI = 10 which show mild to moderate degenerative changes, mild leucocyte infiltration and mild necrosis. (**D**) Treated mice by phage at MOI = 1 which show mild degenerative changes with minimal leucocyte infiltration and mild necrosis. (**E**) Treated group at MOI = 0.1 which show moderate degenerative changes and mild to moderate leucocytic infiltration

Histological analysis of the inflamed heart tissues also confirmed the previous findings. Histologic changes in the heart tissue were mild to moderate, and leukocyte infiltration was mild compared to the positive (infected) control (Fig. 9).

## Discussion

Antibiotic resistance has spread widely in recent years, making the treatment of many pathogenic bacteria very difficult [67]. *E. faecalis* is an opportunistic pathogen that causes severe infections such as UTIs, bacteremia, and bacterial endocarditis [17]. In the last few decades, more than 12% of nosocomial infections and around 20–68% increase in mortality rates after bloodstream infections have been caused by *E. faecalis* [68]. As contemporary medicine has been unable to deal with this serious problem, phage therapy can be used as an alternative to antibiotics [39]. Phage therapy has been used for enteric and systemic treatment in countries such as Russia, Poland, and Eastern Europe [69, 70]. Regarding the Middle East, to the best of our knowledge, phage therapy has not progressed beyond the research stage [71]. In Western Europe and the U.S., phage therapy has been approved only on a case-by-case basis in emergency situations, in accordance with the Helsinki Declaration (Helsinki, Finland, June 1964) [72]. Several successful treatment cases using phage therapy have been reported in the literature [73–75].

The advantages of phage therapy over traditional antibiotics have been intensively reviewed in literature. Phages offers high specificity, a lower risk of secondary infections, and the ability to self-amplify at the site of infection [38]. Researchers have engineered phages with enhanced capabilities, including controlled lytic activity and a broader host range, to target antimicrobial-resistant bacteria [38]. Additionally, phage display technology has transformed vaccine development by presenting antigens in a highly immunogenic form [76]. In cancer therapy, phages are being explored as potential anti-cancer agents, selectively delivering therapeutic substances to cancer cells while sparing healthy tissue [77]. Furthermore, phages show promise as gene delivery vectors, effectively encapsulating and transferring genetic material to targeted cells [77].

However, despite these advancements, phage therapy faces challenges in clinical applications such as maintaining phage stability remains a major concern. Phages can stimulate immune responses, potentially reducing efficacy. Moreover, phage therapy requires standardized protocols and regulatory frameworks, as well as, the risk of horizontal gene transfer leading to spread antibiotic resistance genes among bacterial populations [38, 72].

The ideal phage for phage therapy should have a broad and specific host range targeting the desired microorganism(s). It should also exhibit strong lytic activity and effectively kill bacteria. Additionally, the phage must be stable across a wide range of pH levels, temperatures, and storage conditions, while maintaining genomic stability. It is highly recommended that the phage does not carry antibiotic resistance genes and that its genome is fully sequenced and annotated [78].

In the present study, a lytic phage named vB_Efa_ZAT1 was isolated from sewage water against MDR *E. faecalis* clinical isolate. Several groups have also reported isolation of *Enterococcus* phages from wastewater, compost and water channels [79–81]. Phage ZAT1 belonged to the *Efquatrovirus* genus, according to morphology and genomic analyses. The in vitro activity of the phage was stable to temperature and pH. The reason for this could be the phage’s adaptation to the somewhat basic pH of the sewage water from which it was separated. The phage demonstrated good alkaline pH stability, but its infectivity was slightly decreased at acidic pH. The phage’s optimum temperature was found to be 37 °C, which can be explained by *E. faecalis* being a normal element of human flora. Calcium ions didn’t show any effect on phage adsorption, attachment, and penetration. *Enterococcus* phages were reported to be very stable and could be maintained for two years at 4 °C [82, 83].

All stages of the phage life cycle were visible in one-step growth curve [84]. It was easily possible to determine the phage’s biological parameters such as adsorption rate, latent time and burst size. The adsorption rate was up to nearly 90% within 20 min both in presence and absence of calcium chloride. The latent period was 25 min and studies reported that high phage fitness had optimal latent time attributes [84]. The average burst size was 1263 PFU/cell [79]. The phage showed a comparable latent period as other *E. faecalis* phages, however, with around 3–4 times higher burst size than the highest reported for *E. faecalis* phage HEf13 [85]. The short latent time and the high average burst size indicate the high efficacy of phage ZAT1. The high burst size indicates the high ability of the phage to clear the bacterial cells within a short time. This was also in agreement with the observed plaques which showed bigger size than others [40].

A variety of clinical pathogenic bacterial strains were evaluated as a host for phage ZAT1 and the results showed that the phage was host specific and showed slight activity against certain strain may be due to cytolysin. From this perspective, although the high specificity of the phage is a desired feature to avoid non-specific interaction with beneficial flora, the extremely narrow host range can be considered a limitation. Several studies have investigated phage engineering and the use of phage cocktail to overcome extend the host range [28, 33, 86, 87].

Bacterial biofilms play a key role in pathogenesis and pose a significant challenge for their treatment [88]. In microbial infections where biofilm is formed, antibiotics activity is often limited. *E. faecalis* ability to form biofilms is a critical virulence factor providing resistance to several antibiotics. Therefore, developing new anti-biofilm strategies is essential and phages may be an interesting way of eradicating biofilms and clear infections by working in synergy with antibiotics [89]. The phage’s ability to disseminate biofilms can depend either on depolymerizing enzymes which degrade EPS or on the ability to infect persistent metabolism [81]. Phage ZAT1 was able to significantly reduce the biofilm formation on polystyrene surfaces of MDR *E. faecalis* clinical isolate. Moreover, the viable count was also significantly reduced by the phage.

Several studies described successful phage therapies for *Enterococcus* species [90]. A single IP dose of 3 × 10^8^ PFU injection of the ENB6 phage at 45 min after bacterial challenge was sufficient to rescue 100% of the animals from deadly *E. faecium* induced bacteremia [90]. Administering IME-EF1 (10^10^ PFU) phages 30 min after inoculation with *E. faecalis* led to only 60% therapeutic effectiveness and survival [91], and 100% of the septic BALB/c mice were rescued with phage ΦEF24C (> 10^8^ PFU) [78, 92]. In the present study, a single tiny dose of phage ZAT1 (10^8^ PFU) successfully protected all infected mice against bacteremia induced by VRE (Table 2). It was a phage function, not a generic immune response activation that allowed phage to save bacteremic animals.

**Table 2:** Comparison of phage ZAT1 to other enterococcus phages published in literature

Phage	Stability		Latent period (min)	Burst size (PFU/cell)	Host range (Spectrum)	Anti-biofilm Activity	Ex vivo and in vivo Analysis	Ref
	pH	Temp.						
SFQ1	5–11	50 °C	10	110	Broad	It effectively disrupts the biofilms formed by *E. faecalis*	Not determined	[95]
KEN04	3–11	-80 °C–37 °C	20	138.5	Broad	Significantly disrupted biofilms and reduced the bacterial population. It could not inhibit the biofilm formation	Not determined	[96]
HEf13	5–12	4–60 °C	25	352	Broad	Not determined	Effective against human dentin ex vivo infection model	[85]
IME-EF1	Not determined	Not determined	25	60	Moderate	Not determined	60% survival when administered 30 min after infection and 40% survival when administered 4 h after infection	[91]
φEF24C	--	--	30	110	Broad	Not determined	100% survival when administered 20 min after infection at MOI 0.01	[78, 92]
SRG1	3–11	37–65 °C	21	512	Narrow	Not determined	Not determined	[79]
SA14	6–9	4 °C -75°	20	20	Narrow	1.3 log cycles reduction after 2 h at MOI of 100 and reduced bacterial population	Not determined	[40]
ZAT1	5–10	4–65 °C	25	1263	Narrow	90% biofilm reduction and reduced bacterial population	100% survival when administered 1 h after infection at MOI of 0.1	This study

All mice were successfully rescued from the lethal bacteremia throughout the entire experimental period, except when a phage solution with a MOI of 10 was administered. In that case, one out of seven mice died (a 15% mortality rate). This outcome could be due to several factors that warrant further investigation. One possibility is the development of bacterial resistance to the phage; however, this was unlikely, as the bacterial count in the mice’s blood was significantly lower than in the control groups (MOI = 0) and phage-treated group (MOI = 1 and MOI = 0.1), indicating effective bacterial killing. Another potential explanation could be an overstimulation of the immune system due to the high phage dose or the presence of trace levels of endotoxins and exotoxins in the phage preparations.

Nevertheless, the use of phage ZAT1 in combating *E. faecalis* infections is limited by its narrow spectrum, the inadequate information about its pharmacokinetics and the host immune response. Further investigation of these subjects is highly recommended for successful introduction to the medical field. Therefore, some organizations are forcing towards the implementation of phage therapy in health sectors as a potential alternative [93, 94]. Until then, phage therapy will remain the last resort for difficult to treat bacterial infections as demonstrated is a number of clinical studies [73, 93].

## Conclusions

Phages can be a safe and effective alternative to traditional antibiotics for the treatment of *E. faecalis* infection. Phage use appears to be a promising strategy for reducing the biofilm bacterial load associated with *E. faecalis* infections. The present work adds to the growing evidence that the phage therapy potential is worth pursuing. Phage therapy however encounters clinical hurdles, encompassing phage stability and dissemination, production costs, regulatory ambiguities, immune responses, bacterial resistance, scalability, quality control, public perception, and acceptance [97]. Key strategies to boost phage therapy: tailored approaches, combo therapies, nanoparticle delivery, engineered phages, efficient trials, regulation, public education and cost-effectiveness [90, 98].

## Electronic supplementary material

Below is the link to the electronic supplementary material.

Additional file 1 (**Table **S1: Summary of animal specification used for in vivo study, **Table S2**: Annotation and functional analysis of ZAT1 genome, and **Table S3**: Genomic sequence similarly of ZAT1 with other phages deposited in NCBI).

## Data Availability

The phage vB_Efa_ZAT1 full genome sequence was deposited in the NCBI GenBank database under accession number PP438801.

## References

[1] Tenover FC. Mechanisms of antimicrobial resistance in bacteria. Am J Med. 2006;119:S3–10. 16735149 10.1016/j.amjmed.2006.03.011

[2] Pereira LB, Zanetti MOB, Sponchiado LP, Rodrigues JPV, Campos MS, de Varallo A. FR, Antibiotic use in Brazilian hospitals in the 21st century: a systematic review. Rev Soc Bras Med Trop. 2021;54. 10.1590/0037-8682-0861-2020 PMC8282254 34133621

[3] Polishchuck NM, Matylonok TY, Kyryk DL, Alimenko YL. Molecular aspects of bacterial resistance to antibiotics. Ann Mechnikov’s Inst. 2021;2:6–11.

[4] Murray CJ, Ikuta KS, Sharara F, Swetschinski L, Robles Aguilar G, Gray A, et al. Global burden of bacterial antimicrobial resistance in 2019: a systematic analysis. Lancet. 2022;399:629–55. 35065702 10.1016/S0140-6736(21)02724-0 PMC8841637

[5] Jaja IF, Jaja C-JI, Chigor NV, Anyanwu MU, Maduabuchi EK, Oguttu JW, et al. Antimicrobial resistance phenotype of *Staphylococcus aureus* and *Escherichia coli* isolates obtained from meat in the formal and informal sectors in South Africa. Biomed Res Int. 2020;2020:3979482. 33015163 10.1155/2020/3979482 PMC7525293

[6] Chai WC, Whittall JJ, Song D, Polyak SW, Ogunniyi AD, Wang Y, et al. Antimicrobial action and reversal of resistance in MRSA by difluorobenzamide derivatives targeted at FtsZ. Antibiotics. 2020;9:873. 33291418 10.3390/antibiotics9120873 PMC7762090

[7] Wilson DN, Hauryliuk V, Atkinson GC, O’Neill AJ. Target protection as a key antibiotic resistance mechanism. Nat Rev Microbiol. 2020;18:637–48. 32587401 10.1038/s41579-020-0386-z

[8] Angst DC, Tepekule B, Sun L, Bogos B, Bonhoeffer S. Comparing treatment strategies to reduce antibiotic resistance in an *in vitro* epidemiological setting. Proc Natl Acad Sci. 2021;118:e2023467118. 10.1073/pnas.2023467118 PMC8020770 33766914

[9] Ibargüen-Mondragón E, Gómez MC, Burbano-Rosero EM. Assessing the role of bacterial plasmid replication in a competition model of sensitive and resistant bacteria to antibiotics. AIMS Math. 2021;6:9446–67.

[10] Beomidehagh M, Rezaee MA, Ganbarov K, Jafari F, Hasani A, Alizadeh N, et al. Effect of acidic and alkali shocks on expression of efaA gene in *Enterococcus faecalis*, isolated from root canal infection. Cell Mol Biol. 2018;64:1–5. 30403587

[11] Chenoweth C, Schaberg D. The epidemiology of enterococci. Eur J Clin Microbiol Infect Dis. 1990;9:80–9. 2180711 10.1007/BF01963631

[12] Weiner-Lastinger LM, Abner S, Edwards JR, Kallen AJ, Karlsson M, Magill SS, et al. Antimicrobial-resistant pathogens associated with adult healthcare-associated infections: Summary of data reported to the National Healthcare Safety Network, 2015–2017. Infect Control Hosp Epidemiol. 2020;41:1–18. 31767041 10.1017/ice.2019.296 PMC8276252

[13] Oravcova V, Mihalcin M, Zakova J, Pospisilova L, Masarikova M, Literak I. Vancomycin-resistant enterococci with *vanA* gene in treated municipal wastewater and their association with human hospital strains. Sci Total Environ. 2017;609:633–43. 28763660 10.1016/j.scitotenv.2017.07.121

[14] Pandey RP, Mukherjee R, Chang C-M. Antimicrobial Resistance Surveillance System Mapping in Different Countries. Preprints; 2022 [cited 2022 Aug 7]; Available from: https://www.preprints.org/manuscript/202201.0234/v1 10.33393/dti.2022.2482 PMC9714473 36479338

[15] Dowd SE, Sun Y, Secor PR, Rhoads DD, Wolcott BM, James GA, et al. Survey of bacterial diversity in chronic wounds using pyrosequencing, DGGE, and full ribosome shotgun sequencing. BMC Microbiol. 2008;8:43. 18325110 10.1186/1471-2180-8-43 PMC2289825

[16] Bowler PG, Duerden BI, Armstrong DG. Wound microbiology and associated approaches to wound management. Clin Microbiol Rev. 2001;14:244–69. 11292638 10.1128/CMR.14.2.244-269.2001 PMC88973

[17] Chong KKL, Tay WH, Janela B, Yong AMH, Liew TH, Madden L, et al. *Enterococcus faecalis* modulates immune activation and slows healing during wound infection. J Infect Dis. 2017;216:1644–54. 29045678 10.1093/infdis/jix541 PMC5854026

[18] WHO. Global guidelines for the prevention of surgical site infection. World Health Organization; 2016. 27929621

[19] Willems RJ, van Schaik W. Transition of *Enterococcus faecium* from commensal organism to nosocomial pathogen. Future Microbiol. 2009;4:1125–35. 19895216 10.2217/fmb.09.82

[20] van Harten RM, Willems RJL, Martin NI, Hendrickx APA. Multidrug-resistant enterococcal infections: new compounds, novel antimicrobial therapies? Trends Microbiol. 2017;25:467–79. 28209400 10.1016/j.tim.2017.01.004

[21] Yu W, Zhang J, Tong J, Zhang L, Zhan Y, Huang Y, et al. *In vitro* antimicrobial activity of fosfomVancomycinomycin and daptomycin alone, and in combination, against linezolid-resistant *Enterococcus faecalis*. Infect Dis Ther. 2020;9:927–34. 32964392 10.1007/s40121-020-00342-1 PMC7680468

[22] Ono S, Muratani T, Matsumoto T. Mechanisms of resistance to imipenem and ampicillin in *Enterococcus faecalis*. Antimicrob Agents Chemother. 2005;49:2954–8. 15980374 10.1128/AAC.49.7.2954-2958.2005 PMC1168717

[23] Dale JL, Nilson JL, Barnes AMT, Dunny GM. Restructuring of *Enterococcus faecalis* biofilm architecture in response to antibiotic-induced stress. Npj Biofilms Microbiomes. 2017;3:15. 28685097 10.1038/s41522-017-0023-4 PMC5493694

[24] Khan A, Davlieva M, Panesso D, Rincon S, Miller WR, Diaz L, et al. Antimicrobial sensing coupled with cell membrane remodeling mediates antibiotic resistance and virulence in *Enterococcus faecalis*. Proc Natl Acad Sci. 2019;116:26925–32. 31818937 10.1073/pnas.1916037116 PMC6936494

[25] Hollenbeck BL, Rice LB. Intrinsic and acquired resistance mechanisms in *Enterococcus*. Virulence. 2012;3:421–569. 23076243 10.4161/viru.21282 PMC3485979

[26] Keen EC. Phage therapy: concept to cure. Front Microbiol. 2012;3:238. 22833738 10.3389/fmicb.2012.00238 PMC3400130

[27] Lam HYP, Lai M-J, Chen T-Y, Wu W-J, Peng S-Y, Chang K-C. Therapeutic effect of a newly isolated lytic bacteriophage against multi-drug-resistant *Cutibacterium acnes* infection in mice. Int J Mol Sci. 2021;22:7031. 34209998 10.3390/ijms22137031 PMC8268795

[28] Malik S, Nehra K, Rana JS. Bacteriophage cocktail and phage antibiotic synergism as promising alternatives to conventional antibiotics for the control of multi-drug-resistant uropathogenic *Escherichia coli*. Virus Res. 2021;198496. 10.1016/j.virusres.2021.198496 34182014

[29] Nale JY, Clokie MRJ. Preclinical data and safety assessment of phage therapy in humans. Curr Opin Biotechnol. 2021;68:310–7. 33862490 10.1016/j.copbio.2021.03.002 PMC8150739

[30] Dutta DS, Sarkar DR, Bordoloi DKP, Deka DC, Sonowal DPJ. Bacteriophage therapy to combat antibiotic resistance: a brief review. Pharma Innov J. 2021;10:389–94.

[31] Holger D, Kebriaei R, Morrisette T, Lev K, Alexander J, Rybak M. Clinical pharmacology of bacteriophage therapy: a focus on multidrug-resistant *Pseudomonas aeruginosa* infections. Antibiotics. 2021;10:556. 34064648 10.3390/antibiotics10050556 PMC8151982

[32] Squires RA. Bacteriophage therapy for challenging bacterial infections: achievements, limitations and prospects for future clinical use by veterinary dermatologists. Vet Dermatol. 2021;32:587–e158. 33870572 10.1111/vde.12958

[33] Loc-Carrillo C, Abedon ST. Pros and cons of phage therapy. Bacteriophage. 2011;1:111–4. 22334867 10.4161/bact.1.2.14590 PMC3278648

[34] Parasion S, Kwiatek M, Gryko R, Mizak L, Malm A. Bacteriophages as an alternative strategy for fighting biofilm development. Pol J Microbiol. 2014;63:137–45. 25115107

[35] Song M, Wu D, Hu Y, Luo H, Li G. Characterization of an Enterococcus faecalis bacteriophage vB_EfaM_LG1 and its synergistic Effect with Antibiotic. Front Cell Infect Microbiol. 2021;11:698807. 34336721 10.3389/fcimb.2021.698807 PMC8322680

[36] Holger DJ, Lev KL, Kebriaei R, Morrisette T, Shah R, Alexander J, et al. Bacteriophage-antibiotic combination therapy for multidrug-resistant Pseudomonas aeruginosa: In vitro synergy testing. J Appl Microbiol. 2022;133:1636–49. 10.1111/jam.15647 35652690

[37] Kunz Coyne AJ, Eshaya M, Bleick C, Vader S, Biswas B, Wilson M, et al. Exploring synergistic and antagonistic interactions in phage-antibiotic combinations against ESKAPE pathogens. Microbiol Spectr. 2024;12:10. 10.1128/spectrum.00427-24 PMC11468199 39082827

[38] Abdelkader K, Gerstmans H, Saafan A, Dishisha T, Briers Y. The preclinical and clinical progress of bacteriophages and their lytic enzymes: the parts are easier than the whole. Viruses. 2019;11:96. 30678377 10.3390/v11020096 PMC6409994

[39] Kortright KE, Chan BK, Koff JL, Turner PE. Phage therapy: a renewed approach to combat antibiotic-resistant bacteria. Cell Host Microbe. 2019;25:219–32. 30763536 10.1016/j.chom.2019.01.014

[40] Ali Z, Dishisha T, El-Gendy AO, Azmy AF. Isolation and phenotypic characterization of bacteriophage SA14 with lytic-and anti-biofilm activity against multidrug-resistant *Enterococcus faecalis*. Beni-Suef Univ J Basic Appl Sci. 2023;12:1–10.

[41] Molham F, Khairalla AS, Azmy AF, El-Gebaly E, El-Gendy AO, AbdelGhani S. Anti-proliferative and anti-biofilm potentials of bacteriocins produced by non-pathogenic *Enterococcus* sp. Probiotics Antimicrob Proteins. 2021;13:571–85. 33010007 10.1007/s12602-020-09711-1

[42] Mattila S, Ruotsalainen P, Jalasvuori M. On-Demand isolation of bacteriophages against drug-resistant bacteria for personalized phage therapy. Front Microbiol. 2015;6:1271. 26617601 10.3389/fmicb.2015.01271 PMC4643220

[43] Kropinski AM, Mazzocco A, Waddell TE, Lingohr E, Johnson RP. Enumeration of bacteriophages by double agar overlay plaque assay BT - bacteriophages: methods and protocols, 1: isolation, characterization, and interactions. Methods Mol Biol Humana Press. 2009;501:69–76. 10.1007/978-1-60327-164-6_7 19066811

[44] Obradović M, Malešević M, Di Luca M, Kekić D, Gajić I, et al. Isolation, characterization, genome analysis and host resistance development of two novel *Lastavirus* phages active against pandrug-resistant *Klebsiella pneumoniae*. Viruses. 2023;15:628. 36992337 10.3390/v15030628 PMC10052179

[45] Abdelkader K, Gutiérrez D, Grimon D, Ruas-Madiedo P, Lood C, Lavigne R, et al. Lysin LysMK34 of *Acinetobacter baumannii* bacteriophage PMK34 has a turgor pressure-dependent intrinsic antibacterial activity and reverts colistin resistance. Appl Environ Microbiol. 2020;86:e01311–20. 32709718 10.1128/AEM.01311-20 PMC7499038

[46] Latka A, Drulis-Kawa Z. Advantages and limitations of microtiter biofilm assays in the model of antibiofilm activity of *Klebsiella* phage KP34 and its depolymerase. Sci Rep. 2020;10:20338. 33230270 10.1038/s41598-020-77198-5 PMC7683578

[47] Peng F, Mi Z, Huang Y, Yuan X, Niu W, et al. Characterization, sequencing and comparative genomic analysis of vB_AbaM-IME-AB2, a novel lytic bacteriophage that infects multidrug-resistant *Acinetobacter baumannii* clinical isolates. BMC Microbiol. 2014;14:181. 24996449 10.1186/1471-2180-14-181 PMC4094691

[48] Choi S-K, Jeong H, Kloepper JW, Ryu C-M. Genome sequence of *Bacillus amyloliquefaciens* GB03, an active ingredient of the first commercial biological control product. Genome Announc. 2014;2:e01092–14. 25359911 10.1128/genomeA.01092-14 PMC4214987

[49] Seemann T. Prokka: rapid prokaryotic genome annotation. Bioinformatics. 2014;30:2068–9. 24642063 10.1093/bioinformatics/btu153

[50] Delcher AL, Bratke KA, Powers EC, Salzberg SL. Identifying bacterial genes and endosymbiont DNA with glimmer. Bioinformatics. 2007;23:673–9. 17237039 10.1093/bioinformatics/btm009 PMC2387122

[51] Janeček Š, Zámocká B. A new GH13 subfamily represented by the α-amylase from the halophilic archaeon *Haloarcula hispanica*. Extremophiles. 2020;24:207–17. 31734852 10.1007/s00792-019-01147-y

[52] Marchler-Bauer A, Anderson JB, Chitsaz F, Derbyshire MK, DeWeese-Scott C, Fong JH, et al. CDD: specific functional annotation with the conserved domain database. Nucleic Acids Res. 2009;37:D205–10. 18984618 10.1093/nar/gkn845 PMC2686570

[53] Mitchell AL, Attwood TK, Babbitt PC, Blum M, Bork P, Bridge A, et al. InterPro in 2019: improving coverage, classification and access to protein sequence annotations. Nucleic Acids Res. 2019;47:D351–60. 30398656 10.1093/nar/gky1100 PMC6323941

[54] Tan D, Svenningsen S, Lo, Middelboe M. Quorum sensing determines the choice of antiphage defense strategy in *Vibrio anguillarum*. MBio. 2015;6:e00627–15. 26081633 10.1128/mBio.00627-15 PMC4471561

[55] Hallgren J, Tsirigos KD, Pedersen MD, Almagro Armenteros JJ, Marcatili P, Nielsen H, et al. DeepTMHMM predicts alpha and beta transmembrane proteins using deep neural networks. BioRxiv. 2022;2022(04):08–487609.

[56] Lowe TM, Chan PP. tRNAscan-SE On-line: integrating search and context for analysis of transfer RNA genes. Nucleic Acids Res. 2016;44:W54–7. 27174935 10.1093/nar/gkw413 PMC4987944

[57] Grant JR, Stothard P. The CGView server: a comparative genomics tool for circular genomes. Nucleic Acids Res. 2008;36:W181–4. 18411202 10.1093/nar/gkn179 PMC2447734

[58] Yukgehnaish K, Rajandas H, Parimannan S, Manickam R, Marimuthu K, Petersen B, et al. PhageLeads: rapid assessment of phage therapeutic suitability using an ensemble machine learning approach. Viruses. 2022;14:342. 35215934 10.3390/v14020342 PMC8879740

[59] Richter M, Rosselló-Móra R, Oliver Glöckner F, Peplies J. JSpeciesWS: a web server for prokaryotic species circumscription based on pairwise genome comparison. Bioinformatics. 2016;32:929–31. 26576653 10.1093/bioinformatics/btv681 PMC5939971

[60] Moraru C, Varsani A, Kropinski AM. VIRIDIC - A novel tool to calculate the intergenomic similarities of prokaryote-infecting viruses. Viruses. 2020;12:1268. 33172115 10.3390/v12111268 PMC7694805

[61] Nishimura Y, Yoshida T, Kuronishi M, Uehara H, Ogata H, Goto S. ViPTree: the viral proteomic tree server. Bioinformatics. 2017;33:2379–80. 28379287 10.1093/bioinformatics/btx157

[62] Chhibber S, Kumari S. Application of therapeutic phages in medicine. In Bacteriophages. InTech; Ipek Kurtboke Ed., 2012.

[63] Hesse S, Malachowa N, Porter AR, Freedman B, Kobayashi SD, Gardner DJ, et al. Bacteriophage treatment rescues mice infected with multidrug-resistant *Klebsiella pneumoniae* ST258. MBio. 2021;12:e00034–21. 33622728 10.1128/mBio.00034-21 PMC8545083

[64] Kumari S, Harjai K, Chhibber S. Efficacy of bacteriophage treatment in murine burn wound infection induced by *Klebsiella pneumoniae*. J Microbiol Biotechnol. 2009;19:622–8. 19597322 10.4014/jmb.0808.493

[65] Laboratory of Chemical Life Science, Center B. Institute for Chemical Research, Kyoto University J. DigAlign: the Dynamic Genomic Alignment Server, version 2.0. Available from: https://www.genome.jp/digalign/

[66] Seby R, Kim C, Khreis M, Khreis K. *Enterococcus faecalis*-induced infective endocarditis: an unusual source of infection and a rare clinical presentation. J Int Med Res. 2022;50:03000605221112019. 35899534 10.1177/03000605221112019 PMC9340997

[67] Church NA, McKillip JL. Antibiotic resistance crisis: challenges and imperatives. Biol (Bratisl). 2021;76:1535–50.

[68] Martone WJ. Spread of Vancomycin-resistant enterococci: why did it happen in the United States? Infect Control Hosp Epidemiol. 1998;19:539–45. 9758052 10.1086/647870

[69] Haq IU, Chaudhry WN, Akhtar MN, Andleeb S, Qadri I. Bacteriophages and their implications on future biotechnology: a review. Virol J. 2012;9:1–8. 22234269 10.1186/1743-422X-9-9 PMC3398332

[70] Moore SA, Moore AY. Phage therapy. Overcoming Antimicrob Resist Ski. 2021;195.

[71] Alsaadi A, Imam M, Alghamdi AA, Alghoribi MF. Towards promising antimicrobial alternatives: the future of bacteriophage research and development in Saudi Arabia. J Infect Public Health. 2022;15:1355–62. 36332378 10.1016/j.jiph.2022.10.022

[72] McCallin S, Sacher JC, Zheng J, Chan BK. Current state of compassionate phage therapy. Viruses. 2019;11:343. 31013833 10.3390/v11040343 PMC6521059

[73] Dedrick RM, Guerrero-Bustamante CA, Garlena RA, Russel DA, Ford K, et al. Engineered bacteriophages for treatment of a patient with a disseminated drug-resistant *Mycobacterium abscessus*. Nat Med. 2019;25:730–3. 31068712 10.1038/s41591-019-0437-z PMC6557439

[74] Dedrick RM, Smith BE, Cristinziano M, Freeman KG, Jacobs-Sera D, Belessis Y, et al. Phage therapy of *Mycobacterium* infections: compassionate use of phages in 20 patients with drug-resistant mycobacterial disease. Clin Infect Dis. 2023;76:103–12. 35676823 10.1093/cid/ciac453 PMC9825826

[75] Aslam S, Courtwright AM, Koval C, Lehman SM, Morales S, Furr CLL, et al. Early clinical experience of bacteriophage therapy in 3 lung transplant recipients. Am J Transpl. 2019;19:2631–9. 10.1111/ajt.15503 PMC6711787 31207123

[76] Cui L, Watanabe S, Miyanaga K, Kiga K, Sasahara T, Aiba Y, et al. A comprehensive review on phage therapy and phage-based drug development. Antibiot. 2024;13:870. 10.3390/antibiotics13090870 PMC11428490 39335043

[77] Islam MS, Fan J, Pan F. The power of phages: revolutionizing cancer treatment. Front Oncol. 2023;13:1290296. 38033486 10.3389/fonc.2023.1290296 PMC10684691

[78] Uchiyama J, Rashel M, Takemura I, Wakiguchi H, Matsuzaki S. In silico and in vivo evaluation of bacteriophage φEF24C, a candidate for treatment of Enterococcus faecalis infections. Appl Environ Microbiol. 2008;74:4149–63. 18456848 10.1128/AEM.02371-07 PMC2446516

[79] Rahmat Ullah S, Andleeb S, Raza T, Jamal M, Mehmood K. Effectiveness of a lytic phage SRG1 against vancomycin-resistant *Enterococcus faecalis* in compost and soil. BioMed Res Int. 2017; 2017:9351017. 10.1155/2017/9351017 PMC5632989 29147662

[80] Otawa K, Hirakata Y, Kaku M, Nakai Y. Bacteriophage control of Vancomycin-resistant *Enterococci* in cattle compost. J Appl Microbiol. 2012;113:449–507. 10.1111/j.1365-2672.2012.05361.x 22702478

[81] Azeredo J, García P, Drulis-Kawa Z. Targeting biofilms using phages and their enzymes. Curr Opin Biotechnol. 2021;68:251–61. 33714050 10.1016/j.copbio.2021.02.002

[82] Raza T, Andleeb S, Ullah SR, Jamal M, Mehmood K, Ali M. Isolation and characterization of a phage to control Vancomycin resistant *Enterococcus faecium*. Open Life Sci. 2018;13:553–60. 33817126 10.1515/biol-2018-0066 PMC7874677

[83] Paul K, Merabishvili M, Hazan R, Christner M, Herden U, Gelman D, et al. Bacteriophage rescue therapy of a Vancomycin-resistant *Enterococcus faecium* infection in a one-year-old child following a third liver transplantation. Viruses. 2021;13:1785. 34578366 10.3390/v13091785 PMC8472888

[84] Wang I-N. Lysis timing and bacteriophage fitness. Genetics. 2006;172:17–26. 16219778 10.1534/genetics.105.045922 PMC1456144

[85] Lee D, Im J, Na H, Ryu S, Yun CH, Han SH. The novel *Enterococcus* phage vB_EfaS_HEf13 has broad lytic activity against clinical isolates of *Enterococcus faecalis*. Front Microbiol. 2019;10:496990. 10.3389/fmicb.2019.02877 PMC6927925 31921055

[86] Yoichi M, Abe M, Miyanaga K, Unno H, Tanji Y. Alteration of tail fiber protein gp38 enables T2 phage to infect *Escherichia coli* O157:H7. J Biotechnol. 2005;115:101–7. 15607229 10.1016/j.jbiotec.2004.08.003

[87] Mahichi F, Synnott AJ, Yamamichi K, Osada T, Tanji Y. Site-specific recombination of T2 phage using IP008 long tail fiber genes provides a targeted method for expanding host range while retaining lytic activity. FEMS Microbiol Lett. 2009;295:211–7. 19453513 10.1111/j.1574-6968.2009.01588.x

[88] Chaudhry WN, Haq IU, Andleeb S, Qadri I. Characterization of a virulent bacteriophage LK1 specific for *Citrobacter freundii* isolated from sewage water. J Basic Microbiol. 2014;54:531–41. 23686910 10.1002/jobm.201200710

[89] Abedon ST, Danis-Wlodarczyk KM, Wozniak DJ, Sullivan MB. Improving phage-biofilm *in vitro* experimentation. Viruses. 2021;13:1175. 34205417 10.3390/v13061175 PMC8234374

[90] Biswas B, Adhya S, Washart P, Paul B, Trostel AN, Powell B, et al. Bacteriophage therapy rescues mice bacteremic from a clinical isolate of Vancomycin-resistant *Enterococcus faecium*. Infect Immun. 2002;70:204–10. 11748184 10.1128/IAI.70.1.204-210.2002 PMC127648

[91] Zhang W, Mi Z, Yin X, Fan H, An X, Zhang Z, et al. Characterization of *Enterococcus faecalis* phage IME-EF1 and its endolysin. PLoS ONE. 2013;8:e80435. 24236180 10.1371/journal.pone.0080435 PMC3827423

[92] Uchiyama J, Rashel M, Maeda Y, Takemura I, Sugihara S, Akechi K, et al. Isolation and characterization of a novel *Enterococcus faecalis* bacteriophage φEF24C as a therapeutic candidate. FEMS Microbiol Lett. 2008;278:200–6. 18096017 10.1111/j.1574-6968.2007.00996.x

[93] Djebara S, Maussen C, De Vos D, Merabishvili M, Damanet B, Pang KW, et al. Processing phage therapy requests in a Brussels Military Hospital: lessons identified. Viruses. 2019;11:265. 30884879 10.3390/v11030265 PMC6466067

[94] Scottish Health Technologies Group– Health Improvement Scotland. SHTG recommendations, in response to enquiries from a clinical bacteriophage specialist. February 2023, Available at: https://shtg.scot/media/2325/20230113-shtg-recommendation-for-bacteriophage-therapy-v10.pdf

[95] Song F, Sheng J, Tan J, Xie H, Wang X, Guo W. Characterization of an *Enterococcus faecalis* bacteriophage SFQ1 as a potential therapeutic agent. Front Microbiol. 2023;14:1210319. 37426023 10.3389/fmicb.2023.1210319 PMC10324664

[96] Soro O, Kigen C, Nyerere A, Gachoya M, Georges M, Odoyo E, et al. Characterization and anti-biofilm activity of lytic *Enterococcus* phage vB_Efs8_KEN04 against clinical isolates of multidrug-resistant *Enterococcus faecalis* in Kenya. Viruses. 2024;16:1275. 39205249 10.3390/v16081275 PMC11360260

[97] Curtright AJ, Abedon ST. Phage therapy: emergent property pharmacology. J Bioanal Biomed. 2011;4:1–14.

[98] Sulakvelidze A, Morris JG. Bacteriophages as therapeutic agents. Ann Med. 2001;33:507–9. 11730156 10.3109/07853890108995959

