# Supplementary material for: Therapeutic potential of a newly isolated bacteriophage against multi-drug resistant Enterococcus faecalis infections: in vitro and in vivo characterization
Source: BMC Microbiol. 2025 Feb 20;25:80. doi: 10.1186/s12866-025-03785-z (PMC11841226; doi:10.1186/s12866-025-03785-z)
Supplement: Supplementary file 1 — Additional file 1 (Table S1: Summary of animal specification used for in vivo study, Table S2: Annotation and functional analysis of ZAT1 genome, and Table S3: Genomic sequence similarly of ZAT1 with other phages deposited in NCBI). [file 12866_2025_3785_MOESM1_ESM.docx]

Electronic Supplementary Information

Therapeutic potential of a newly isolated bacteriophage against multi-drug resistant *Enterococcus faecalis* infections: *in vitro* and *in vivo* characterization

*Zienab Ali,^a^ Karim Abdelkader,^a^ Maha M. Abdel-Fattah,^a,b^ Ahmed Farag Azmy,^a^ Ahmed O. El-Gendy,^a^ Tarek Dishisha^a^*

*^a^Department of Pharmaceutical Microbiology and Immunology, Faculty of Pharmacy, Beni-Suef University, 62511 Beni-Suef, Egypt*

*^b^Department of Pharmacology and Toxicology, Faculty of Pharmacy, Beni-Suef University, 62511 Beni-Suef, Egypt*

Corresponding author:

E-mail: [Tarek.Dishisha@pharm.bsu.edu.eg](mailto:Tarek.Dishisha@pharm.bsu.edu.eg)

**Table S1.** Summary of animal specification used for *in vivo* study.

| **Items for comparison** | **Determination of LD100% of MDR *E. faecalis*** | **Treatment by ZAT-1 phage** |
| --- | --- | --- |
| **Weight** | 22-30 g | 22-30 g |
| **Gender** | Male | Male |
| **No. of groups** | 6 | 6 |
| **No. of mice in each group** | 5 | 6 |
| **Groups name** | 10^2^, 10^4^, 10^6^, 10^8^, 10^9^ and 10^10^ | Positive, negative control,  Phage only, (MOI = 10), (MOI = 1), (MOI = 0.1). |
| **Doses** | 100 µl | 200 µL |
| **Sampling interval** | - | 1, 3, 5, 12, 24 and 72 hr |

Table S2. Annotation of the ZAT1 genome and functional analysis of the detected *orf*s using BLASTx ^1^ analysis against non-redundant data base and search for conserved domains ^2^. Predicted function was assigned according to the best hit (highest identity and coverage).

|  | **Start (nt)** | **End (nt)** | **Length (nt)** | **Start codon** | **aa** | **Putative function** | **Closest hit (E-value)** | **Identity%**  **(coverage %)** | **Accession No.** | **Conserved domain**  **(E-value)** |
| --- | --- | --- | --- | --- | --- | --- | --- | --- | --- | --- |
| orf 1 | 31 | 354 | 324 | ATG | 107 | Hypothetical protein | hypothetical protein FDI49_gp19 [*Enterococcus* phage phiSHEF4]  (3e-43) | 97.3  (67) | YP_009613308.1 |  |
| orf 2 | 475 | 978 | 504 | ATG | 167 | Hypothetical protein | hypothetical protein FDI49_gp19 [*Enterococcus* phage phiSHEF4]  (5e-25) | 97  (29) | YP_009613367.1 |  |
| orf 3 | 1022 | 1318 | 297 | ATG | 98 | Head-tail connector protein | Head-tail connector protein [*Enterococcus* phage phiSHEF2]  (5e-64) | 98.9  (98) | YP_009613289.1 | Gp6_gp15 like superfamily  (1.01e-06) |
| orf 4 | 1290 | 1625 | 336 | ATG | 111 | Head-tail adaptor protein | head-tail adaptor protein [*Enterococcus* phage vB_EfaS-271]  (4e-74) | 97.30  (99) | QLF85382.1 | Phage H_T join superfamily  (4.95e-04) |
| orf 5 | 1622 | 2029 | 408 | ATG | 135 | Head tail joining protein | head-tail joining protein  [*Enterococcus* phage EFRM31]  (7e-96) | 100  (99) | YP_004306649.1 | phage protein, HK97 gp10 family  (3.11e-05) |
| orf 6 | 2026 | 2391 | 366 | ATG | 121 | Head tail joining protein | head-tail joining protein  [*Enterococcus* phage EFRM31]  (3e-83) | 99  (99) | YP_004306650.1 |  |
| orf 7 | 2467 | 3033 | 567 | ATG | 188 | Tail major protein | putative major tail tube protein [*Enterococcus* phage EfaCPT1]  (2e-129) | 99.5  (99) | YP_009103828.1 | Phage_TPP_1 superfamily  (4.68e-08) |
| orf 8 | 3231 | 3542 | 312 | ATG | 103 | Tail tape measure chaperone protein | Putative tail tape measure chaperone protein [*Enterococcus* phage EFA1]  (3e-67) | 100  (99) | QOI58196.1 |  |
| orf 9 | 3799 | 8169 | 4371 | ATG | 1456 | Tail length tape-measure protein | Tail length tape-measure protein [*Enterococcus* phage SANTOR1]  (0.0) | 96.7  (99) | YP_009284725.1 | lyz_like_superfamily  (1.57e-15) |
| orf 10 | 8251 | 10335 | 2085 | ATG | 694 | Tail family protein | Tail family protein [*Enterococcus* phage vB_EfaS-SRH2]  (0.0) | 93  (99) | BCU01272.1 | Ser S superfamily  (8.19e-03) |
| orf 11 | 10406 | 12463 | 2058 | ATG | 685 | endopeptidase tail protein | Tail assembly protein [*Enterococcus* phage vB_EfaS_IME196]  (0.0) | 95  (99) | YP_009216604.1 | Prophage tail superfamily  (3.08e-04) |
| orf 12 | 12469 | 12765 | 297 | ATG | 98 | Hypothetical protein | hypothetical protein [*Enterococcus* phage vB_EfaS-SRH2] | 95  (86) | BCU01270.1 |  |
| orf 13 | 12943 | 13188 | 246 | ATG | 81 | Tail fiber protein | Tail fiber protein [*Enterococcus* phage Ec-ZZ2]  (1e-25) | 98  (76) | YP_009303721.1 |  |
| orf 14 | 13203 | 13436 | 234 | ATG | 77 | Holin | holin [*Enterococcus* phage vB_EfaS_AL3]  (8e-45) | 100  (98) | YP_009624747.1 | Phage holin superfamily  (2.01e-20) |
| orf 15 | 13439 | 14422 | 984 | ATG | 327 | Endolysin | N-acetylmuramoyl-L-alanine amidase family protein [*Enterococcus* phage AUEF3]  (0.0) | 99  (99) | YP_009625865.1 | Amidase _2 (interval of 145-522; 3.73e-03)  ZoocinA_TRD (interval of 673-978; 1.59e-20) |
| orf 16 | 14513 | 14740 | 228 | ATG | 75 | glutaredoxin | putative glutaredoxin [*Enterococcus* phage vB_EfaS_Paulomi]  (8e-45) | 93  (98) | UGO52513.1 | Thioredoxin like superfamily  (1.91e-09) |
| orf 17 | 14760 | 17051 | 2292 | ATG | 763 | DNA polymerase B | putative DNA polymerase B region protein [*Enterococcus* phage vB_EfaS_Paulomi]  (0.0) | 97.38  (99) | UGO52514.1 | POLBc_Pol_II_B  (1.75e-05) |
| orf 18 | 17087 | 17818 | 732 | ATG | 243 | DNA adenine methylase | DNA methylase [*Enterococcus* phage vB_EfaS_Max]  (9e-159) | 88.5  (99) | QAX97263.1 | Mehtyltransf D12 superfamily  (1.67e-04) |
| orf 19 | 17854 | 18078 | 225 | ATG | 74 | Hypothetical protein | hypothetical protein FDI50_gp46 [*Enterococcus* phage phiSHEF5]  (9e-46) | 100  (98) | YP_009613457.1 |  |
| orf 20 | 18149 | 18910 | 762 | ATG | 253 | Hypothetical protein | hypothetical protein EfaCPT1_gp24 [*Enterococcus* phage EfaCPT1]  (0.0) | 99.2  (99) | YP_009103841.1 |  |
| orf 21 | 19183 | 18932 | 252 | ATG | 83 | Hypothetical protein | hypothetical protein AVT93_gp26 [*Enterococcus* phage vB_EfaS_IME196]  (4e-51) | 100  (98) | YP_009216613.1 |  |
| orf 22 | 19184 | 19480 | 297 | ATG | 98 | Hypothetical protein | hypothetical protein FDI49_gp19 [*Enterococcus* phage phiSHEF4]  (1e-63) | 98  (98) | YP_009613367.1 |  |
| orf 23 | 19481 | 20299 | 819 | ATG | 272 | Hypothetical protein | DUF1351 protein [*Enterococcus* phage MSF2]  (0.0) | 100  (98) | QDF15359.1 | DUF1351  (3.83e-15) |
| orf 24 | 20453 | 21229 | 777 | ATG | 258 | Hypothetical protein | hypothetical protein [*Enterococcus* phage vB_EfaS_Ef5.1]  (0.0) | 97.29  (99) | QBZ69901.1 | - |
| orf 25 | 21240 | 21719 | 480 | ATG | 159 | homing endonucleases | hypothetical protein [*Enterococcus* faecalis]  (2e-113) | 99.37  (99) | WP_240208213.1 | NUMOD4 motif  (0.0) |
| orf 26 | 21716 | 21922 | 207 | ATG | 68 | Hypothetical protein | hypothetical protein FDI49_gp25 [*Enterococcus* phage phiSHEF4]  (2e-114) | 98.2  (81) | YP_009613373.1 |  |
| orf 27 | 21924 | 22523 | 600 | ATG | 199 | Hypothetical protein | hypothetical protein FDI50_gp37 [*Enterococcus* phage phiSHEF5]  (4e-139) | 96  (99) | YP_009613448.1 | DUF3310  (1.26e-10) |
| orf 28 | 22680 | 22877 | 198 | ATG | 65 | Hypothetical protein | hypothetical protein vBEfaS271_36 [*Enterococcus* phage vB_EfaS-271]  (1e-40) | 100  (98) | QLF85405.1 |  |
| orf 29 | 22879 | 23622 | 744 | TTG | 247 | DNA polymerase | Bi-functional DNA primase/polymerase [*Enterococcus* phage vB_EfaS_LM99]  (1e-160) | 87  (99) | AXC33926.1 | - Prim-Pol superfamily (6.82e-06)  -Primase C terminal 1 (PriCT-1, 1.2e-10) |
| orf 30 | 23603 | 24136 | 534 | ATG | 177 | HNH homing endonuclease | HNH homing endonuclease [*Enterococcus* phage vB_EfaS_Ef5.1]  (2e-107) | 93.2  (99) | QBZ69907.1 | HNH_3  (5.3e-10) |
| orf 31 | 24148 | 24342 | 195 | ATG | 64 | Hypothetical protein | hypothetical protein [*Enterococcus* phage EFap02]  (3e-35) | 95.3  (98) | UIE13731.1 |  |
| orf 32 | 24354 | 24542 | 189 | ATG | 62 | Hypothetical protein | hypothetical protein FDI50_gp34 [*Enterococcus* phage phiSHEF5]  (1e-35) | 100  (98) | YP_009613445.1 |  |
| orf 33 | 24556 | 24753 | 198 | ATG | 65 | Hypothetical protein | hypothetical protein PAULFLAKE_38 [*Enterococcus* phage vB_EfaS_Paulo]  (8e-40) | 100  (98) | UGO52529.1 | PgpA  (0.0) |
| orf 34 | 24813 | 24989 | 177 | ATG | 58 | Hypothetical protein | hypothetical protein FDI49_gp30 [*Enterococcus* phage phiSHEF4]  (5e-32) | 100  (98) | YP_009613378.1 |  |
| orf 35 | 24986 | 26281 | 1296 | ATG | 431 | DNA helicase | DNA helicase [*Enterococcus* phage vB_EfaS-SRH2]  (0.0) | 97  (100) | BCU01248.1 | SNF2-rel_dom (6.4e-10)  Helicase_C (8.0e-06) |
| orf 36 | 26274 | 26660 | 387 | ATG | 128 | Endonuclease | endonuclease [*Enterococcus* phage IME-EF4]  (1e-66) | 96.9  (99) | YP_009004334.1 |  |
| orf 37 | 26660 | 26866 | 207 | ATG | 68 | Hypothetical protein | hypothetical protein CH14_gp22 [*Enterococcus* phage IME-EF4]  (3e-39) | 100  (98) | YP_009004333.1 |  |
| orf 38 | 26868 | 27029 | 162 | ATG | 53 | Hypothetical protein | hypothetical protein FDI48_gp45 [*Enterococcus* phage phiSHEF2]  (1e-26) | 98.1  (98) | YP_009613325.1 |  |
| orf 39 | 27031 | 27303 | 273 | ATG | 90 | Hypothetical protein | hypothetical protein FDJ59_gp33 [Enterococcus phage AUEF3]  (6e-41) | 95.9  (80) | YP_009625889.1 |  |
| orf 40 | 27352 | 27795 | 444 | ATG | 147 | Hypothetical protein | hypothetical protein TV217_46 [*Enterococcus* phage vB_EFaS_TV217]  (6e-101) | 97.3  (99) | QMS41996.1 | PHA02264  (9.14e-08) |
| orf 41 | 27868 | 28056 | 189 | ATG | 62 | Hypothetical protein | hypothetical protein EfaCPT1_gp45 [*Enterococcus* phage EfaCPT1]  (1e-34) | 100  (98) | YP_009103862.1 |  |
| orf 42 | 28135 | 29727 | 1593 | ATG | 530 | Primase | DNA primase [Enterococcus phage PMBT2] (0.0) | 99  (99) | YP_009622371.1 | COG3378 superfamily  (1.72e-21) |
| orf 43 | 29823 | 30020 | 198 | ATG | 65 | Hypothetical protein | hypothetical protein [*Enterococcus* phage vB_EfaS-SRH2]  (6e-25) | 96.9  (98) | QOI58204.1 | - |
| orf 44 | 30017 | 30256 | 240 | ATG | 79 | Hypothetical protein | hypothetical protein [*Enterococcus* phage EFA1]  (4e-42) | 95.8  (98) | QOI58204.1 |  |
| orf 45 | 30253 | 30471 | 219 | GTG | 72 | Hypothetical protein | hypothetical protein EfaCPT1_gp52 [*Enterococcus* phage EfaCPT1]  (1e-43) | 95.8  (98) | YP_009103869.1 |  |
| orf 46 | 30471 | 30692 | 222 | ATG | 73 | Hypothetical protein | hypothetical protein PAULFLAKE_55 [*Enterococcus* phage vB_EfaS_Paulom | 98.6  (98) | UGO52546.1 |  |
| orf 47 | 30692 | 30832 | 141 | ATG | 46 | Hypothetical protein | hypothetical protein [*Enterococcus* phage EFA1]  (6e-25) | 95.6  (97) | QOI58230.1 |  |
| orf 48 | 31010 | 31273 | 264 | ATG | 87 | Hypothetical protein | hypothetical protein CPT_Sigurd_062 [*Enterococcus* phage Sigurd]  (3e-39) | 74.1  (98) | QYW02460.1 |  |
| orf 49 | 31385 | 31669 | 285 | ATG | 94 | Hypothetical protein | hypothetical protein [*Enterococcus* phage vB_EfaS_Ef6.4]  (1e-44) | 79.5  (97) | QBZ69360.1 |  |
| orf 50 | 31681 | 32010 | 330 | ATG | 109 | Hypothetical protein | hypothetical protein [*Enterococcus* phage vB_EfaS_Ef6.1]  (3e-59) | 92.5  (85) | QBZ69164.1 |  |
| orf 51 | 32007 | 32231 | 225 | ATG | 74 | Hypothetical protein | hypothetical protein [*Enterococcus* phage vB_EfaS_Ef5.1]  (8e-41) | 94.6  (98) | QBZ69936.1 |  |
| orf 52 | 32244 | 32687 | 444 | ATG | 147 | Hypothetical protein | hypothetical protein TV54_64 [*Enterococcus* phage vB_EFaS_TV54]  (2e-101) | 97.3  (99) | QNL31391.1 |  |
| orf 53 | 33287 | 33490 | 204 | ATG | 67 | Hypothetical protein | hypothetical protein BI091_gp58 [*Enterococcus* phage SANTOR1]  (1e-30) | 83.6  (98) | YP_009284769.1 |  |
| orf 54 | 33573 | 33779 | 207 | ATG | 68 | Hypothetical protein | hypothetical protein TV217_66 [*Enterococcus* phage vB_EFaS_TV217]  (8e-29) | 91.2  (98) | QMS42016.1 |  |
| orf 55 | 33779 | 34150 | 372 | ATG | 123 | HNH Homo-endonuclease | homing endonuclease [*Enterococcus* phage vB_EfaS_Ef5.3]  (2e-86) | 98.4  (99) | QBZ70290.1 | McrA endonuclease  (8.03e-03) |
| orf 56 | 34545 | 34697 | 153 | ATG | 50 | Hypothetical protein | hypothetical protein EFAP1_gp22 [*Enterococcus* phage EFAP-1]  (4e-28) | 100  (98) | YP_002727894.1 |  |
| orf 57 | 34712 | 35047 | 336 | ATG | 111 | Endo-deoxyribonuclease | endodeoxyribonuclease [*Enterococcus* phage IME_EF3]  (6e-71) | 96  (99) | YP_009008900.1 | MSCRAMM -SdrC  Superfamily  (2.03e-03) |
| orf 58 | 35050 | 35523 | 474 | ATG | 157 | Terminase small subunit | terminase small-subunit [*Enterococcus* phage EFAP-1]  (6e-71) | 99.4  (99) | YP_002727893.1 | Terminase_4 superfamily  (1.72e-06) |
| orf 59 | 36058 | 37782 | 1725 | ATG | 574 | Terminase large subunit | terminase large subunit [*Enterococcus* phage phiNASRA1]  (0.0) | 99.5  (99) | QEL53368.1 | Terminase_1 superfamily  (1.41e-28) |
| orf 60 | 37871 | 38137 | 267 | ATG | 88 | Hypothetical protein | hypothetical protein [*Enterococcus* phage vB_EfaS_159]  (2e-41) | 73.9  (98) | CAD0310420.1 | Gpbc  (8.98e-03) |
| orf 61 | 38214 | 38378 | 165 | ATG | 54 | Hypothetical protein | hypothetical protein BI091_gp02 [*Enterococcus* phage SANTOR1]  (1e-16) | 94.4  (98) | YP_009284713.1 |  |
| orf 62 | 38383 | 39534 | 1152 | ATG | 383 | Portal protein | portal protein [*Enterococcus* phage phiSHEF5]  (0.0) | 99.7  (99) | YP_009613475.1 | Phage portal superfamily  (1.93e-16) |
| orf 63 | 39521 | 40084 | 564 | GTG | 187 | Phage maturation protease | head maturation protease [*Enterococcus* phage vB_EfaS_IME196]  (5e-122) | 98.9  (99) | YP_009216592.1 | Peptidase_S78  (9.08e-17) |
| orf 64 | 40154 | 41407 | 1254 | ATG | 417 | Major head protein | Major head protein [*Enterococcus* phage PMBT2]  (0.0) | 98.8  (99) | YP_009622331.1 | phage major capsid protein, HK97 superfamily  (2.32e-12) |
| orf 65 | 41533 | 41733 | 201 | ATG | 66 | Major tail protein | Major tail protein [*Enterococcus* phage IME-EF4]  (1e-36) | 98.5  (98) | YP_009004365.1 |  |
| orf 66 | 41777 | 42073 | 297 | ATG | 98 | head-tail adaptor | head-tail adaptor Ad1 [*Enterococcus* phage phiSHEF2]  (2e-63) | 98  (98) | YP_009613289.1 | Gp9_gp15 like superfamily  (1.86e-04) |
| orf 67 | 42045 | 42455 | 411 | ATG | 136 | Head closure Hc1 | head closure Hc1 [*Enterococcus* phage phiSHEF5]  (2e-63) | 94.2  (75) | YP_009613470.1 | Phage H_T_superfamily  (3.92e-03) |

**Table S3.** Genomic sequence similarly of *Enterococcus* phage vB_Efa-ZAT1 with other phages deposited in NCBI. The listed phages are selected based on BLASTn analysis of *Enterococcus* phage vB_Efa-ZAT1 assembled genomic sequence.

| **Phage scientific name** | **Genome size** | **Genus** | **Query cover (%)** | **Similarity (%)** | **E. value** | **Accession number** |
| --- | --- | --- | --- | --- | --- | --- |
| *Enterococcus phage Ef212, complete genome* | 40690 | *Efquatrovirus* | 91% | 92.14 | 0.0 | [OR052631.1](https://www.ncbi.nlm.nih.gov/nuccore/OR052631.1/) |
| *Enterococcus* phage phiSHEF2, complete genome | 41712 | *Efquatrovirus* | 88% | 91.49% | 0.0 | [NC_042021.1](https://www.ncbi.nlm.nih.gov/nucleotide/NC_042021.1?report=genbank&log$=nucltop&blast_rank=1&RID=YF87VMG401R) |
| *Enterococcus* phage phiSHEF10, complete genome | 41680 | *Efquatrovirus* | 87% | 91.49% | 0.0 | [OL799256.1](https://www.ncbi.nlm.nih.gov/nucleotide/OL799256.1?report=genbank&log$=nucltop&blast_rank=2&RID=YF87VMG401R) |
| *Enterococcus* phage vB_EfaS_Max, complete genome | 40975 | *Efquatrovirus* | 89% | 91.38% | 0.0 | [MK360024.1](https://www.ncbi.nlm.nih.gov/nucleotide/MK360024.1?report=genbank&log$=nucltop&blast_rank=3&RID=YF87VMG401R) |
| *Enterococcus* phage vB_EFaS_TV217, complete genome | 41486 | *Efquatrovirus* | 89% | 91.18% | 0.0 | [MT627482.1](https://www.ncbi.nlm.nih.gov/nucleotide/MT627482.1?report=genbank&log$=nucltop&blast_rank=4&RID=YF87VMG401R) |
| *Enterococcus* phage PMBT2, complete genome | 41489 | *Efquatrovirus* | 88% | 88.89% | 0.0 | [NC_042101.1](https://www.ncbi.nlm.nih.gov/nucleotide/NC_042101.1?report=genbank&log$=nucltop&blast_rank=5&RID=YF87VMG401R) |
| *Enterococcus* phage vB_EFaS_TV51, complete genome | 41821 | *Efquatrovirus* | 87% | 88.44% | 0.0 | [MT661597.1](https://www.ncbi.nlm.nih.gov/nucleotide/MT661597.1?report=genbank&log$=nucltop&blast_rank=6&RID=YF87VMG401R) |
| *Enterococcus* phage vB_EfaS_Ef5.2, complete genome | 41418 | *Efquatrovirus* | 85% | 88.35% | 0.0 | [MK721186.1](https://www.ncbi.nlm.nih.gov/nucleotide/MK721186.1?report=genbank&log$=nucltop&blast_rank=7&RID=YF87VMG401R) |
| *Enterococcus* phage vB_EFaS_TV54, complete genome | 42438 | *Efquatrovirus* | 86% | 88.06% | 0.0 | [MT661598.1](https://www.ncbi.nlm.nih.gov/nucleotide/MT661598.1?report=genbank&log$=nucltop&blast_rank=8&RID=YF87VMG401R) |
| *Enterococcus* phage vB_EfaS_Ef5.3, complete genome | 39115 | *Efquatrovirus* | 83% | 89.16% | 0.0 | [MK721200.1](https://www.ncbi.nlm.nih.gov/nucleotide/MK721200.1?report=genbank&log$=nucltop&blast_rank=9&RID=YF87VMG401R) |
| *Enterococcus* phage vB_EfaS_Ef5.1, complete genome | 41141 | *Efquatrovirus* | 86% | 88.86% | 0.0 | [MK721199.1](https://www.ncbi.nlm.nih.gov/nucleotide/MK721199.1?report=genbank&log$=nucltop&blast_rank=10&RID=YF87VMG401R) |
| *Enterococcus* phage EfaCPT1, complete genome | 40923 | *Efquatrovirus* | 88% | 87.67% | 0.0 | [NC_025465.1](https://www.ncbi.nlm.nih.gov/nucleotide/NC_025465.1?report=genbank&log$=nucltop&blast_rank=11&RID=YF87VMG401R) |
| *Enterococcus* phage EfaCPT1, complete genome | 40923 | *Efquatrovirus* | 88% | 87.67% | 0.0 | [JX193904.1](https://www.ncbi.nlm.nih.gov/nucleotide/JX193904.1?report=genbank&log$=nucltop&blast_rank=12&RID=YF87VMG401R) |
| *Enterococcus* phage AUEF3, partial genome | 41257 | *Efquatrovirus* | 87% | 91.05% | 0.0 | [NC_042134.1](https://www.ncbi.nlm.nih.gov/nucleotide/NC_042134.1?report=genbank&log$=nucltop&blast_rank=13&RID=YF87VMG401R) |
| *Enterococcus* phage vB_EfaS_Ef5.4, complete genome | 40685 | *Efquatrovirus* | 87% | 87.83% | 0.0 | [MK721191.1](https://www.ncbi.nlm.nih.gov/nucleotide/MK721191.1?report=genbank&log$=nucltop&blast_rank=14&RID=YF87VMG401R) |
| *Enterococcus* phage vB_EfaS-SRH2 DNA, complete sequence | 21115 | *Efquatrovirus* | 84% | 85.99% | 0.0 | [NC_012419.1](https://www.ncbi.nlm.nih.gov/nucleotide/NC_012419.1?report=genbank&log$=nucltop&blast_rank=15&RID=YF87VMG401R) |
| *Enterococcus* phage SANTOR1, complete genome | 38749 | *Efquatrovirus* | 65% | 94.06% | 0.0 | [LC623721.1](https://www.ncbi.nlm.nih.gov/nucleotide/LC623721.1?report=genbank&log$=nucltop&blast_rank=16&RID=YF87VMG401R) |
| *Enterococcus* phage SANTOR1, complete genome | 37933 | *Efquatrovirus* | 65% | 93.60% | 0.0 | [KX284704.1](https://www.ncbi.nlm.nih.gov/nucleotide/KX284704.1?report=genbank&log$=nucltop&blast_rank=17&RID=YF87VMG401R) |
| *Enterococcus* phage vB_EfaS_LM99, complete genome | 37933 | *Efquatrovirus* | 78% | 93.60% | 0.0 | [NC_031051.1](https://www.ncbi.nlm.nih.gov/nucleotide/NC_031051.1?report=genbank&log$=nucltop&blast_rank=18&RID=YF87VMG401R) |
| *Enterococcus* phage vB_EfaS_Paulomi, complete genome | 40203 | *Efquatrovirus* | 80% | 93.43% | 0.0 | [MH355583.1](https://www.ncbi.nlm.nih.gov/nucleotide/MH355583.1?report=genbank&log$=nucltop&blast_rank=19&RID=YF87VMG401R) |
| *Enterococcus* phage phiSHEF11, complete genome | 41921 | *Efquatrovirus* | 74% | 93.14% | 0.0 | [OL539449.1](https://www.ncbi.nlm.nih.gov/nucleotide/OL539449.1?report=genbank&log$=nucltop&blast_rank=20&RID=YF87VMG401R) |
| *Enterococcus* phage phiSHEF5, complete genome | 40790 | *Efquatrovirus* | 77% | 92.17% | 0.0 | [OL799257.1](https://www.ncbi.nlm.nih.gov/nucleotide/OL799257.1?report=genbank&log$=nucltop&blast_rank=21&RID=YF87VMG401R) |
| *Enterococcus* phage vB_OCPT_CCS4, complete genome | 41598 | *Efquatrovirus* | 74% | 92.15% | 0.0 | [NC_042023.1](https://www.ncbi.nlm.nih.gov/nucleotide/NC_042023.1?report=genbank&log$=nucltop&blast_rank=22&RID=YF87VMG401R) |
| *Enterococcus* phage vB_EfaS-271, complete genome | 40050 | *Efquatrovirus* | 75% | 92.05% | 0.0 | [ON113176.1](https://www.ncbi.nlm.nih.gov/nucleotide/ON113176.1?report=genbank&log$=nucltop&blast_rank=23&RID=YF87VMG401R) |
| *Enterococcus* phage EF326P1, complete genome | 40197 | *Efquatrovirus* | 69% | 92.01% | 0.0 | [MT520979.1](https://www.ncbi.nlm.nih.gov/nucleotide/MT520979.1?report=genbank&log$=nucltop&blast_rank=24&RID=YF87VMG401R) |
| *Enterococcus* phage LY0322, complete genome | 41558 | *Efquatrovirus* | 73% | 91.91% | 0.0 | [OP172797.1](https://www.ncbi.nlm.nih.gov/nucleotide/OP172797.1?report=genbank&log$=nucltop&blast_rank=25&RID=YF87VMG401R) |
| *Enterococcus* phage ZXL, complete genome | 40934 | *Efquatrovirus* | 73% | 91.89% | 0.0 | [NC_042125.1](https://www.ncbi.nlm.nih.gov/nucleotide/NC_042125.1?report=genbank&log$=nucltop&blast_rank=26&RID=YF87VMG401R) |
| *Enterococcus* phage vB_EfaS_785CC, complete genome | 40804 | *Efquatrovirus* | 74% | 91.89% | 0.0 | [ON113334.1](https://www.ncbi.nlm.nih.gov/nucleotide/ON113334.1?report=genbank&log$=nucltop&blast_rank=27&RID=YF87VMG401R) |
| *Enterococcus* phage vB_EfaS_785CS, complete genome | 40956 | *Efquatrovirus* | 74% | 91.85% | 0.0 | [MZ272341.1](https://www.ncbi.nlm.nih.gov/nucleotide/MZ272341.1?report=genbank&log$=nucltop&blast_rank=28&RID=YF87VMG401R) |
| *Enterococcus* phage phiSHEF4, complete genome | 40946 | *Efquatrovirus* | 73% | 91.84% | 0.0 | [MZ182246.1](https://www.ncbi.nlm.nih.gov/nucleotide/MZ182246.1?report=genbank&log$=nucltop&blast_rank=29&RID=YF87VMG401R) |
| *Enterococcus* phage vB_EfaS_AL3, complete genome | 41081 | *Efquatrovirus* | 70% | 91.64% | 0.0 | [NC_042022.1](https://www.ncbi.nlm.nih.gov/nucleotide/NC_042022.1?report=genbank&log$=nucltop&blast_rank=30&RID=YF87VMG401R) |
| *Enterococcus* phage EFA1, complete genome | 40789 | *Efquatrovirus* | 76% | 91.59% | 0.0 | [NC_042126.1](https://www.ncbi.nlm.nih.gov/nucleotide/NC_042126.1?report=genbank&log$=nucltop&blast_rank=31&RID=YF87VMG401R) |
| *Enterococcus* phage vB_EfS_L1, complete genome | 40454 | *Efquatrovirus* | 71% | 91.48% | 0.0 | [MT857001.1](https://www.ncbi.nlm.nih.gov/nucleotide/MT857001.1?report=genbank&log$=nucltop&blast_rank=32&RID=YF87VMG401R) |
| *Enterococcus* phage vB_Efa29212_2e, complete genome | 41151 | *Efquatrovirus* | 78% | 90.72% | 0.0 | [OP254195.1](https://www.ncbi.nlm.nih.gov/nucleotide/OP254195.1?report=genbank&log$=nucltop&blast_rank=33&RID=YF87VMG401R) |
| *Enterococcus* phage vB_EfaS_IME196, complete genome | 41351 | *Efquatrovirus* | 80% | 91.17% | 0.0 | [OP559177.1](https://www.ncbi.nlm.nih.gov/nucleotide/OP559177.1?report=genbank&log$=nucltop&blast_rank=34&RID=YF87VMG401R) |
| *Enterococcus* phage vB_EfaS_IME196, complete genome | 38886 | *Efquatrovirus* | 80% | 88.87% | 0.0 | [KT932701.1](https://www.ncbi.nlm.nih.gov/nucleotide/KT932701.1?report=genbank&log$=nucltop&blast_rank=35&RID=YF87VMG401R) |
| *Enterococcus* phage phiNASRA1, complete genome | 38886 | *Efquatrovirus* | 73% | 88.87% | 0.0 | [NC_028990.1](https://www.ncbi.nlm.nih.gov/nucleotide/NC_028990.1?report=genbank&log$=nucltop&blast_rank=36&RID=YF87VMG401R) |
| *Enterococcus* phage Ec-ZZ2, complete genome | 40139 | *Efquatrovirus* | 71% | 93.36% | 0.0 | [BK022041.1](https://www.ncbi.nlm.nih.gov/nucleotide/BK022041.1?report=genbank&log$=nucltop&blast_rank=37&RID=YF87VMG401R) |
| *Enterococcus* phage Ec-ZZ2, complete genome | 41170 | *Efquatrovirus* | 71% | 94.29% | 0.0 | [MG264739.2](https://www.ncbi.nlm.nih.gov/nucleotide/MG264739.2?report=genbank&log$=nucltop&blast_rank=38&RID=YF87VMG401R) |
| *Enterococcus* phage FX417, complete genome | 41170 | *Efquatrovirus* | 72% | 87.66% | 0.0 | [BK024197.1](https://www.ncbi.nlm.nih.gov/nucleotide/BK024197.1?report=genbank&log$=nucltop&blast_rank=39&RID=YF87VMG401R) |
| *Enterococcus* phage vB_EfaS_AL2, complete genome | 41061 | *Efquatrovirus* | 67% | 95.97% | 0.0 | [KR131750.1](https://www.ncbi.nlm.nih.gov/nucleotide/KR131750.1?report=genbank&log$=nucltop&blast_rank=40&RID=YF87VMG401R) |
| *Enterococcus* phage IME-EF4, complete genome | 40836 | *Efquatrovirus* | 70% | 95.97% | 0.0 | [NC_031260.1](https://www.ncbi.nlm.nih.gov/nucleotide/NC_031260.1?report=genbank&log$=nucltop&blast_rank=41&RID=YF87VMG401R) |
| *Enterococcus* phage IME-EF4, complete genome | 40692 | *Efquatrovirus* | 70% | 95.88% | 0.0 | [NC_015270.1](https://www.ncbi.nlm.nih.gov/nucleotide/NC_015270.1?report=genbank&log$=nucltop&blast_rank=42&RID=YF87VMG401R) |
| *Enterococcus* phage LY0323, complete genome | 40692 | *Efquatrovirus* | 72% | 95.88% | 0.0 | [GU815339.1](https://www.ncbi.nlm.nih.gov/nucleotide/GU815339.1?report=genbank&log$=nucltop&blast_rank=43&RID=YF87VMG401R) |
| *Enterococcus* phage MSF2, complete genome | 40876 | *Efquatrovirus* | 67% | 95.83% | 0.0 | [MT829326.1](https://www.ncbi.nlm.nih.gov/nucleotide/MT829326.1?report=genbank&log$=nucltop&blast_rank=44&RID=YF87VMG401R) |
| *Enterococcus* phage vB_EfaS_Ef6.1, complete genome | 40880 | *Efquatrovirus* | 67% | 95.83% | 0.0 | [NC_042127.1](https://www.ncbi.nlm.nih.gov/nucleotide/NC_042127.1?report=genbank&log$=nucltop&blast_rank=45&RID=YF87VMG401R) |
| *Enterococcus* phage vB_EfaS_Ef6.1, complete genome | 41871 | *Efquatrovirus* | 70% | 95.80% | 0.0 | [NC_023551.1](https://www.ncbi.nlm.nih.gov/nucleotide/NC_023551.1?report=genbank&log$=nucltop&blast_rank=46&RID=YF87VMG401R) |
| *Enterococcus* phage Sigurd, complete genome | 40429 | *Efquatrovirus* | 66% | 95.80% | 0.0 | [KF733017.1](https://www.ncbi.nlm.nih.gov/nucleotide/KF733017.1?report=genbank&log$=nucltop&blast_rank=47&RID=YF87VMG401R) |
| *Enterococcus* phage IME_EF3, complete genome | 41811 | *Efquatrovirus* | 76% | 95.61% | 0.0 | [MH375074.1](https://www.ncbi.nlm.nih.gov/nucleotide/MH375074.1?report=genbank&log$=nucltop&blast_rank=48&RID=YF87VMG401R) |
| *Enterococcus* phage IME_EF3, complete genome | 41687 | *Efquatrovirus* | 76% | 92.44% | 0.0 | [MK982307.1](https://www.ncbi.nlm.nih.gov/nucleotide/MK982307.1?report=genbank&log$=nucltop&blast_rank=49&RID=YF87VMG401R) |
| *Enterococcus* phage vB_EfaS_Ef6.4, complete genome | 41687 | *Efquatrovirus* | 63% | 90.95% | 0.0 | [OP889240.1](https://www.ncbi.nlm.nih.gov/nucleotide/OP889240.1?report=genbank&log$=nucltop&blast_rank=50&RID=YF87VMG401R) |
| *Enterococcus* phage EFap02, complete genome | 41276 | *Efquatrovirus* | 62% | 90.36% | 0.0 | [MK721187.1](https://www.ncbi.nlm.nih.gov/nucleotide/MK721187.1?report=genbank&log$=nucltop&blast_rank=51&RID=YF87VMG401R) |

References

1. Altschul SF, Gish W, Miller W, Myers EW, Lipman DJ. Basic local alignment search tool. *J Mol Biol*. 1990;215(3):403-410. doi:https://doi.org/10.1016/S0022-2836(05)80360-2

2. Marchler-Bauer A, Anderson JB, Chitsaz F, et al. CDD: specific functional annotation with the Conserved Domain Database. *Nucleic Acids Res*. 2009;37(suppl_1):D205-D210.
